# Cell-type-resolved mRNA 3′-end remodeling in Alzheimer’s disease

**DOI:** 10.21203/rs.3.rs-11143166/v1

**Published:** 2026-10-07

**Authors:** Yeon Choi, Jaroslav Bendl, Gabriel E. Hoffman, James M. Vicari, Prashant N. M., Roman Kosoy, Xinyi Wang, Seon Shmuel Kinrot, Hui Yang, Kiran Girdhar, Donghoon Lee, John F. Fullard, Pengfei Dong, Panos Roussos

**Affiliations:** 1 Center for Disease Neurogenomics, Icahn School of Medicine at Mount Sinai, New York, NY, USA; 2 Friedman Brain Institute, Icahn School of Medicine at Mount Sinai, New York, NY, USA; 3 Department of Psychiatry, Icahn School of Medicine at Mount Sinai, New York, NY, USA; 4 Department of Genetics and Genomic Sciences, Icahn School of Medicine at Mount Sinai, New York, NY, USA; 5 Center for Precision Medicine and Translational Therapeutics, James J. Peters VA Medical Center, Bronx, NY, USA; 6 Mental Illness Research, Education and Clinical Center VISN2, James J. Peters VA Medical Center, Bronx, NY, USA

## Abstract

Alternative polyadenylation diversifies messenger RNAs by altering coding sequences and 3′ untranslated regions, but its organization across human brain cell types and disruption in neurodegeneration remain poorly understood. By analyzing 3′-capture single-nucleus RNA sequencing data of 6.3 million nuclei from the prefrontal cortex of 1,494 human donors, we defined 108,768 polyadenylation sites across 25,274 genes, most of which were absent from the GENCODE polyadenylation-site annotation. Polyadenylation-site usage was strongly cell-type-specific and revealed regulatory differences not captured by gene expression. With increasing Alzheimer’s disease clinicopathological severity, excitatory neurons, oligodendrocytes and immune cells showed increased distal-site preference, including reduced relative usage of intronic sites and increased distal 3′ untranslated region usage in synaptic and RNA-processing genes. External single-nucleus data supported neuronal and oligodendrocyte patterns, while complementary analyses of purified microglia supported selected immune-cell findings. Integrative analyses of cleavage and polyadenylation factor expression, co-regulated usage networks, sequence motifs and published perturbations prioritized the cleavage factor Im complex as a candidate regulator. These findings identify cell-type-specific messenger RNA 3′-end remodeling as a reproducible dimension of Alzheimer’s disease molecular pathology that is only partly captured by gene-level analyses.

Single-cell transcriptomic atlases have transformed understanding of the human brain by defining where genes are expressed and how their abundance changes across cell types, aging, and disease. Yet these studies generally summarize RNA at the gene level, overlooking regulated variation at the 3′ ends of messenger RNAs (mRNAs). Alternative polyadenylation (APA) directs cleavage at different sites within the same gene, generating transcripts with distinct coding sequences (CDS) or 3′ untranslated regions (UTRs)^1,2^. Selection of intronic or coding-region sites can alter coding potential or produce transcripts subject to mRNA surveillance, whereas tandem 3′ UTR sites change the inclusion of regulatory sequences that control RNA stability, localization and translation^3,4^. This regulation is particularly relevant in the brain, where neurons express unusually long 3′ UTRs and use them to direct transcripts to specialized compartments such as axons, dendrites, and synapses^5,6^. Genetic variation affecting polyadenylation has also been associated with neurological and psychiatric disorders^7,8^. Nevertheless, the organization of 3′-end selection of mRNA across human brain cell types remains incompletely understood.

Alzheimer’s disease (AD) provides an important setting for investigating this incompletely characterized layer of RNA processing. Synaptic dysfunction and loss are closely associated with cognitive decline^9,10^, and large-scale single-cell studies have identified extensive changes in cellular composition and gene expression across disease progression^11–15^. These gene-level analyses, however, cannot determine whether transcripts from the same gene undergo coordinated changes in 3′-end processing. Bulk-tissue studies have reported altered polyadenylation in AD, including changes in 3′ UTR length, but their interpretation is limited by cellular heterogeneity^8,16,17^. It therefore remains unclear which brain cell types undergo polyadenylation remodeling in AD, whether these changes are independent of parent-gene expression, and which components of the 3′-end processing machinery are associated with them.

Here we analyzed 3′-capture single-nucleus RNA sequencing (snRNA-seq) data from the PsychAD cohort to construct a population-scale, cell-type-resolved map of mRNA 3′ ends in the human dorsolateral prefrontal cortex^11,18^. We adapted a compositional framework to quantify relative polyadenylation site usage within each gene, alongside gene-level abundance, and applied it across brain cell types and AD clinicopathological severity. We identified widespread shifts away from intronic cleavage and toward distal 3′ UTR polyadenylation in excitatory neurons, oligodendrocytes and immune cells. These changes affected synaptic and RNA-regulatory programs and replicated across independent human cohorts and sequencing modalities. Multiple lines of evidence converged on the cleavage factor Im (CFIm) complex, particularly *NUDT21*, as a candidate regulator of AD-associated 3′-end remodeling. Together, these findings identify cell-type-specific 3′-end remodeling of mRNA as a reproducible dimension of AD molecular pathology that is largely invisible to conventional gene expression analysis.

## Comprehensive annotation of polyadenylation sites in the human brain

We defined and quantified polyadenylation sites (PAS) using 3′-capture snRNA-seq from 1,494 PsychAD donors^11,18^, spanning the full human lifespan (0–108 years) and encompassing all pathological and clinical AD stages (Fig. 1a). More than 6.3 million nuclei passing quality control were assigned to a unified cell taxonomy. The PsychAD snRNA-seq data were generated using 3′-capturing 10x Genomics chemistry^19^, which enables PAS quantification from existing libraries^20–22^ (Extended Data Fig. 1a). The full cohort was used to construct the PAS reference; cell-type and disease comparisons used the analytical subsets described below.

Building PAS annotations from raw 3′-end read peaks presents two major challenges: (1) missing PAS from underrepresented cell types or populations, and (2) false-positives arising from internal oligo(A) sequences in the transcript^20,23,24^. To address these challenges, we performed PAS discovery in age-by-cell-type strata^25^ and retained sites supported by reference annotations and, where required, sequence-based prediction (Fig. 1b; Extended Data Fig. 1b; Methods). For each group, PAS were retained if they match: (1) manually annotated GENCODE PAS reference, or (2) at least two out of four PAS databases, or (3) one PAS database and a sequence-based PAS prediction by SCAPTURE (Fig. 1b; Extended Data Fig. 1c,d). The incomplete genomic overlap between PAS annotations of each group (Jaccard index: 0.36–0.44; Fig. 1c) supported the use of stratified discovery to improve representation of sites expressed in restricted cell populations.

Next, we merged overlapping PAS from each group, yielding a unified reference of 108,768 PAS across 25,274 genes (Fig. 1b; Methods; **Supplementary Table 1**). Only ~23% matched GENCODE v38 PAS annotation, with nearly half of 3′ UTR PAS and almost all intronic PAS not annotated in GENCODE (Fig. 1d). In line with this, only ~30% of PAS-mapped reads were mapped to GENCODE PAS (Extended Data Fig. 1e), indicating that many PAS detected in the human cortex are not represented in current GENCODE annotations. In our reference, both GENCODE and non-GENCODE PAS exhibited enrichment of the canonical polyadenylation signal (AAUAAA, Fig. 1e) and other PAS-determining sequence motifs at their expected positions relative to the cleavage site^2,26^ (Extended Data Fig. 1f). More than half of genes utilized multiple PAS (mean 4.3, median 2 per gene), while 8,762 genes (~35%) used only one PAS (Extended Data Fig. 1g). Notably, intronic PAS occurs in over 12,000 genes (~48%), and ~2,600 genes (~10%) have 3′ downstream PAS located beyond annotated gene boundaries. The number of PAS per gene was positively correlated with the gene length (Kendall’s *τ* = 0.53), and genes with 3′ downstream PAS tended to be shorter and harbor fewer PAS (Extended Data Fig. 1h,i).

## Within-gene polyadenylation-site diversity marks constrained and disease-relevant genes

We next tested whether PAS diversity is associated with evolutionary constraint and disease relevance. Since the number of PAS correlates with gene length, we assessed gene loss-of-function tolerance using the recently introduced length-independent score S_het_^27^, and additionally stratified genes by length (Fig. 1f). Multi-PAS genes showed significantly higher S_het_ scores compared to single-PAS genes, indicating stronger selective constraint. Moreover, ANOVA analysis showed that both gene length and PAS count can independently improve the prediction of evolutionary constraint metrics S_het_ and pLI^28^ (Extended Data Fig. 1j). These findings suggest that multi-PAS genes are more evolutionarily constrained, even after accounting for gene length. Consistent with this, MAGMA analysis^29^ of brain disorders showed that multi-PAS genes are significantly associated with both neurodegenerative and neuropsychiatric disorders, whereas single-PAS genes and 3′ downstream PAS genes exhibited no such enrichment (Fig. 1g). The association significance remains after adjusting for cell-type-specific expression by conditional MAGMA analysis^30^ (Extended Data Fig. 1k).

Genes with multiple intronic PAS accounted for the largest fraction of the transcriptome in inhibitory neurons (INs), excitatory neurons (ENs), and oligodendrocyte progenitor cells (OPCs), an intermediate fraction in oligodendrocytes (Oligo) and astrocytes (Astro), and the smallest fraction in Immune, Mural, and endothelial (Endo) cells (Fig. 1h). By contrast, no such cell-type differences were observed for genes with multiple 3′ UTR PAS. Given the functional importance and cell type specificities of multi-PAS or intronic-PAS genes, we performed MSigDB^31^ pathway enrichment analysis on gene sets defined by the number of PAS (Fig. 1i). Single-PAS genes were enriched for translation (mostly ribosomal protein genes), secreted proteins, and G protein-coupled receptors, which were also enriched among short genes. In contrast, signaling, developmental, and gene regulatory pathways were enriched in multi-PAS genes. Multi-intron PAS genes and multi-3′ UTR PAS genes showed shared enrichments. Neurodevelopmental terms were more strongly enriched in multi-intron PAS genes, whereas protein catabolic pathways were more strongly enriched in multi-3′ UTR PAS genes.

## Cell type explains the largest measured component of PAS expression variation

To estimate the proportion of expression variation explained by biological and technical factors, we applied variancePartition at both the gene and PAS levels^32,33^ (Fig. 2a). Similar to gene-level results, cell type (median 33.5%) and inter-individual variation (SubID, median 4.8%) were the major sources of PAS variation, and each remaining factor explained less than 1%. Measured technical factors, including pre-sequencing features and snRNA-seq quality control metrics, explained no more variance for PAS than for genes, indicating that PAS quantification is not strongly influenced by technical bias. PAS with very high variance for a given factor were generally variable at the gene level as well (Extended Data Fig. 2a). For example, sex chromosome–encoded genes varied strongly with sex at both the PAS and gene level, and mitochondrial genes showed high variability across tissue sources. Explained variance between PAS and gene levels was more highly correlated for biological than for technical factors (Fig. 2b; Extended Data Fig. 2b), suggesting that biological factors affect gene and PAS expression together, while technical effects act on PAS more independently.

Several genes showed PAS-level cell type variability that was not apparent at the gene level. For instance, the 3′ UTR PAS of kinesin light chain 1 (*KLC1*) showed high variation between neurons and other cell types, while its gene-level expression remained relatively constant (Fig. 2c,d). Given the roles of *KLC1* in axonal transport, synaptic function, and neurodegeneration^34^, *KLC1* illustrates cell-type-associated PAS regulation despite relatively stable gene-level expression. Gene Set Enrichment Analysis of genes with greater cell type variability at the PAS than at the gene level identified the spliceosome, mitochondrial oxidative phosphorylation (OXPHOS), neurodegenerative disorders (AD, Parkinson’s disease, and Huntington’s disease), and ubiquitin-mediated proteolysis as the top-enriched pathways (Fig. 2e). Of note, many OXPHOS genes also have critical roles in these disorders, including the mitochondrial ATP synthase subunit *ATP5F1B*. The expression of its 3′ UTR PAS was ~2-fold higher in neurons, while gene- and upstream PAS-level expression was comparable (Extended Data Fig. 2c,d). To validate cell-type-variable PAS expression in full-length transcripts, we performed long-read cDNA sequencing using Multiplexed Arrays Sequencing^35^ and Nanopore sequencing^36^. Neuron-specific 3′ UTR PAS expression of *KLC1* and *ATP5F1B* was replicated in both long-read datasets (Fig. 2f; Extended Data Fig. 2e–g).

## Polyadenylation-site selection distinguishes major brain cell types

To systematically evaluate gene- and PAS-level expression across major brain cell types, we used a pseudobulk approach^37^ to aggregate nuclei by cell class and examined variation across individuals. Beyond PAS expression, we quantified PAS usage proportions by applying a centered log-ratio (CLR) transformation to PAS counts within each gene^38^, providing a statistically robust compositional framework for APA inference (Methods). Individual PAS expressions were strongly correlated with their parent gene’s log fold changes (Spearman’s *ρ* ~ 0.9), and were also correlated with changes in PAS usage (*ρ* = 0.2–0.5, Fig. 3a). However, PAS-level expression showed lower pairwise correlation across class-level cell types (median *ρ* = 0.55) than gene-level expression (median *ρ* = 0.81; Fig. 3b). Since PAS-level quantification is inherently sparser, technical noise could contribute to this difference. Nevertheless, the lower correlation persisted when restricting the analysis to highly expressed features (log_2_CPM > 5; Extended Data Fig. 3a), suggesting that PAS-level regulation may provide an additional layer of cell-type-specific transcriptome diversity beyond that captured at the gene level.

To characterize directional APA changes, we computed a weighted usage index (WUI) that summarizes the relative preference for proximal or distal PAS; higher WUI values reflect increased usage of distal (3′) PAS (Fig. 3c). WUI can be calculated either from all PAS within a gene to represent the overall 3′-end position of mRNA, or from 3′ UTR-mapped PAS only to represent 3′ UTR length. These two WUI measures were moderately correlated (*ρ* = 0.68, Extended Data Fig. 3b), indicating they capture overlapping but distinct aspects of 3′-end selection. Remarkably, gene-level expression showed modest negative correlations with both all-PAS ΔWUI and 3′ UTR ΔWUI across cell types (Fig. 3a), suggesting that genes highly expressed in each cell type use shorter 3′ UTRs through APA. Global ΔWUI levels were the highest in Oligo, intermediate in neurons, OPC, and Astro, and the lowest in Mural and Immune cells. Endo showed lower WUI for 3′ UTR but intermediate WUI for all PAS (Fig. 3d).

To investigate functional impacts of APA in shaping cell-type-specific transcriptomes, we clustered genes by their 3′ UTR WUI differences across cell types, then identified enriched gene pathways in each cluster (Fig. 3e,f; **Supplementary Table 2**). Consistent with the negative correlation between gene expression and ΔWUI (Fig. 3a), genes with low 3′ UTR ΔWUI in each cell type showed enrichment for cell-type-specific functions. For example, clusters C1–C4 had low ΔWUI in neurons and were enriched for neuronal functions such as ion transport, cell–cell signaling, and sensory perception. Similarly, C5 comprised genes with low ΔWUI predominantly in endothelial cells, consistent with their enrichment for vascular functions such as basement membrane and tube/epithelium development. In contrast, C6 genes had comparable 3′ UTR lengths across all cell types and were enriched for amino acid activation, a housekeeping process. 3′ UTRs of C7 genes were selectively shortened in immune cells and were enriched for regulation of leukocyte degranulation. C9 and C11 had shorter 3′ UTRs in non-neuronal cell types and were enriched for immune-response functions, reflecting the immune roles of glial and vascular cells. C9 was additionally enriched for RNA processing and mitochondrial organization, suggesting that APA differentially regulates RNA processing and mitochondrial organization between neuronal and non-neuronal cells.

Next, we tested whether cell-type-specific PAS overlap with genetic variants for brain disorders. Cell-type-specific PAS, together with cell-type-specific genes and open chromatin regions (OCRs), were subjected to linkage disequilibrium score regression (LDSC) to assess heritability enrichment for multiple brain disorders^39^ (Methods). Cell-type-specific PAS showed heritability enrichment patterns similar to OCRs across disorders, despite the minimal overlap between them (Jaccard index ~0.01) (Fig. 3g, Extended Data Fig. 3c,d). AD and multiple sclerosis heritability was highly enriched in Immune-specific PAS/OCRs, while autism spectrum disorder, major depressive disorder, schizophrenia, and bipolar disorder heritability was strongly enriched in neuron-specific PAS/OCRs. Parkinson’s disease, attention-deficit/hyperactivity disorder, and amyotrophic lateral sclerosis exhibited broader enrichment patterns across cell types, yet PAS and OCRs remained concordant.

## APA distinguishes AD-associated microglia from other microglial states

Brain myeloid cells, including microglia and perivascular macrophages (PVMs), play central roles in AD pathogenesis^40–43^. Using a subtaxonomy of Immune class from our previous study^40^, we identified differentially expressed genes (DEGs), differentially expressed PAS (DE-PAS), and differential PAS usage genes (DUGs) across PVM and three abundant microglial states (homeostatic, Homeo; adaptive, Adapt; AD-associated, ADAM; Extended Data Fig. 4a; **Supplementary Table 3**). Of note, ADAM from the PsychAD was characterized by high expression of *GPNMB* and is highly similar to disease-associated microglia (DAM) described in previous studies^40^.

At our significance thresholds (Methods), most genes with DE-PAS were also DEGs, while only one-third of DUGs were DEGs. Adapt showed the highest number of DEGs, whereas ADAM showed the highest number of DUGs (Fig. 4a), suggesting that ADAM is the most distinct of the tested cell populations at the PAS-level. Consistently, the correlation between PAS expression and usage was the highest in ADAM, while gene-PAS correlation coefficients were similar across all tested cell populations (Extended Data Fig. 4b). Adapt and PVM preferentially used proximal PAS compared to ADAM and Homeo (Fig. 4b,c; Extended Data Fig. 4c). Similar to the class-level analysis, DUGs showed a weak negative correlation between ΔWUI and gene expression across the PVM and microglial states (Extended Data Fig. 4b).

Remarkably, gene ontology (GO) term enrichment analysis revealed that immune cell activation and differentiation functions were shared across DEGs and DUGs, with the highest enrichment in DUGs at ADAM (Extended Data Fig. 4d). For example, in ADAM, NF-κB subunit gene *REL* showed lowered intronic PAS usage, and NF-κB suppressor *KLF2* showed a significantly shortened 3′ UTR (Fig. 4c,d; Extended Data Fig. 4e). Among top ADAM DUGs, we identified upregulated DAM marker *SPP1* and downregulated marker *MS4A6A*. *SPP1*, which enhances synaptic engulfment and is associated with cognitive decline in AD^44–46^, showed higher usage of a distal 3′ UTR PAS and reduced usage of non-coding PAS, potentially increasing coding transcripts and thereby amplifying protein output in synergy with its mRNA upregulation. Additionally, increased distal 3′ UTR PAS usage may alter protein isoform composition, as the proximal 3′ UTR PAS is specific to isoform *SPP1–207*, which lacks internal exons present in other *SPP1* isoforms (Extended Data Fig. 4e). *MS4A6A*, harboring AD risk variants increasing its expression^42^, displayed higher usage of a PAS specific to the shorter transcript *MS4A6A-201* in ADAM.

## Widespread 3′-lengthening of synaptic and RNA-processing genes in AD

To characterize APA in AD, we tested differential gene/PAS expression and differential PAS usage across AD status using pseudobulks for each cell type. Because AD measurements are heterogeneous and highly correlated, we used the first principal component of AD-associated metrics (DxPC1) as a continuous representation of AD clinicopathological severity (Extended Data Fig. 5a, Methods). Compared to binary case-control status, DxPC1-based analyses detected more DEGs, DUGs, and genes with DE-PAS, with higher π_1_ (estimated fraction of differential expression/usage)^47^ (Fig. 5a and Extended Data Fig. 5b,c). The resulting t-statistics were highly correlated with those from CERAD and Braak scores (Extended Data Fig. 5d), indicating that DxPC1 captures transcriptomic variation associated with AD pathology.

The frequency and directionality of APA shifts in AD were cell-type-specific. While AD-associated DEGs were observed in all major cell types, most AD DUGs occurred in EN, Oligo, and Astro cell types (Fig. 5a,b; **Supplementary Table 4**). In contrast, although IN expressed a substantial portion of multi-PAS genes (Fig. 1h), IN exhibited fewer DUGs and lower π_1_ values (Fig. 5a,b; Extended Data Fig. 5c). ΔWUI values along DxPC1 in both all-PAS and 3′ UTR-only analyses showed that EN, Oligo, and Immune cells preferentially used distal PAS in AD, while Astro showed bidirectional changes (Fig. 5b; Extended Data Fig. 5e). Consistently, aggregating PAS usage by genomic region showed increased use of 3′ UTR PAS and decreased use of upstream PAS (5′ UTR, CDS, noncoding exons, and introns) in AD, most prominently in EN (Fig. 5c). The number of differentially used PAS (DU-PAS) per region further supported this pattern, with 3′ UTR regions enriched for upregulated PAS and upstream regions enriched for downregulated PAS in EN, Oligo, and Immune cells (Extended Data Fig. 5f).

Correlations between gene and PAS expression fold changes in AD ranged from *ρ* = 0.5 to 0.8, lower than those observed in cell-type comparisons (*ρ* ~ 0.9). On the other hand, correlations between PAS expression and usage were markedly higher in AD (*ρ* ~ 0.6) than in cell-type comparisons (*ρ* ~ 0.1–0.3), suggesting a greater contribution of APA in shaping the AD transcriptome (Fig. 5d). Top AD DUGs included previously implicated AD-associated genes in their relevant cell types, such as *EGFR* in Astro, *DLG2* and *SMAD4* in EN, and *CX3CR1* and *SPP1* in Immune^40,44–46,48–52^ (Fig. 5b,e; Extended Data Fig. 5g,h). *EGFR*, which is a major AD risk gene^52^, exhibited significantly reduced usage of its most distal PAS (17_3ut) in AD. This particular PAS overlaps with a predicted binding site for miR-144–3p, a miRNA linked to AD, Parkinson’s disease, and depression^53–55^. *DLG2*, encoding a postsynaptic scaffold protein, showed differential usage across multiple intronic PAS in AD EN. *SMAD4*, a TGF-β-activated transcription factor important for neuronal differentiation, exhibited upregulation of both gene expression and 3′ UTR PAS usage, potentially increasing full-length protein isoform levels cooperatively. *CX3CR1* and *SPP1*, known markers of Homeo and DAM respectively^45^, showed gene-level upregulation and increased distal 3′ UTR PAS usage in AD Immune cells. Notably, both genes also exhibited 3′-lengthening in ADAM relative to other myeloid subtypes (Fig. 4c), suggesting that these AD-associated APA changes may be driven by the increased proportion of ADAM in AD brains^40^.

To assess the reproducibility of our AD APA findings, we analyzed external snRNA-seq data from Mathys et al., comprising 406 individuals from the Religious Orders Study and Rush Memory and Aging Project cohort (hereafter, the Mathys dataset)^12^. PAS-level t-statistics in the Mathys dataset correlated positively with those from the PsychAD DxPC1 analysis (Pearson’s *r* = 0.4 and 0.3 for PAS expression and usage, respectively; Extended Data Fig. 5d). In Mathys EN, intronic DU-PAS associated with CERAD showed predominantly decreased usage, whereas 3′ UTR DU-PAS showed predominantly increased usage, recapitulating the directional pattern observed in PsychAD. Across cell types with sufficient DUGs for comparison, ΔWUI values also correlated positively between the two datasets (*ρ* > 0.4 in IN, EN, Oligo, and Astro; Extended Data Fig. 6a,b). We also analyzed two published datasets of purified human microglia, profiled by long-read Iso-seq^56^ and bulk RNA-seq^43^. In both datasets, distal PAS usage of *SPP1* was increased in AD (p < 0.05), similar to PsychAD (Extended Data Fig. 6c,d). Additionally, DaPars^57,58^ analysis of the same bulk RNA-seq data revealed global 3′ UTR lengthening in AD, including both *CX3CR1* (p.adj < 0.05) and *SPP1* (p < 0.05), suggesting that nuclear 3′ UTR lengthening propagates to total mRNA (Extended Data Fig. 6e). The 3′ UTR length changes of DxPC1 3′ UTR DUGs correlated between PsychAD Immune cells and purified microglia (*r* = 0.43; Extended Data Fig. 6f).

Given the limited overlap between DEGs and DUGs (~10% of DEGs in EN; Fig. 5a,f), we asked which functional categories are specifically enriched among DUGs. GO enrichment analysis revealed that terms related to RNA splicing and localization were enriched among AD DUGs, but not DEGs, across EN, Oligo, and Astro (Fig. 5g; Extended Data Fig. 7a). For instance, the SR protein family gene *SRSF10* displayed increased 3′ UTR PAS usage in AD, alongside reduced expression of the PAS matching the short noncoding transcript SRSF10–208 across all three cell types (Extended Data Fig. 7b,c). *CAPRIN1*, which encodes a regulator of subcellular mRNA localization and synaptic function^59,60^, exhibited differential 3′ UTR PAS usage in AD in both EN and Oligo.

Beyond APA changes in genes encoding RNA localization regulators, alterations in 3′ UTR length can directly influence the subcellular distribution of target transcripts themselves^1,2,5^. To explore this possibility in AD, we utilized published subcellular proteomic data targeting the myelin–axon interface to identify proteins enriched at this compartment in both control and AD brains^61^. Of note, genes encoding myelin–axon interface proteins exhibited longer 3′ UTR annotations and higher WUI (Extended Data Fig. 7d,e). Among the 41 proteins that localized to the myelin–axon interface in AD but not in controls, five of the corresponding genes were identified as DUGs in AD Oligo, with four showing 3′ UTR ΔWUI > 0 (Extended Data Fig. 7f). These findings suggest that 3′ UTR lengthening may contribute to the relocalization of specific mRNAs to the myelin–axon interface in AD.

In EN, synapse-related terms were enriched in both AD DUGs and DEGs (Fig. 5g). Despite this shared enrichment, synaptic genes showed limited overlap between AD DUGs and DEGs (10–30%), indicating that synaptic pathways are controlled at both transcriptional and APA levels but through distinct sets of genes. Across both DEG and non-DEG synaptic genes, multiple DUGs exhibited decreased intronic and increased 3′ UTR PAS usage in AD ENs, which may reinforce full-length protein production of those genes (Fig. 5e; Extended Data Fig. 8,9a,b). Among non-DEGs, 3′ UTR up-regulated genes included pre- and postsynaptic markers such as *PPFIA3*, *ATP6V1A*, and *CAMK2B*, as well as genes implicated in amyloid precursor protein (APP) processing, *CLSTN1* and *P2RX7*^62–64^. Increased 3′ UTR usage was also observed in synaptic DEGs previously implicated in AD pathology (upregulated *ADAM10*^52,65^, *PTEN*^66^; downregulated *HOMER1*^67^, *NTRK2*^68,69^), suggesting widespread APA remodeling of synaptic genes in AD. To test whether these usage changes translate into protein-level differences, we examined a published bulk proteome dataset of AD and control brains^70^. Within synapse-related protein co-expression modules, protein levels were broadly reduced in AD, consistent with synaptic and neuronal loss in disease^9,10^. Against this overall reduction, genes with increased mRNA expression or increased 3′ UTR PAS usage in AD ENs showed less pronounced protein decreases than genes without mRNA- or PAS-level changes. Moreover, in a linear model across synapse-module genes, both 3′ UTR usage and mRNA expression changes in AD ENs independently and positively contributed to protein fold changes (*p* = 9.9 × 10^−4^ and 4.8 × 10^−42^, respectively; Extended Data Fig. 9c–e).

## Cleavage and polyadenylation factor expression is altered and correlated with APA in AD

To identify cleavage and polyadenylation (CPA) factors underlying the widespread 3′-lengthening in AD ENs, we examined their differential expression. We focused on 15 core CPA factors from the CPSF, CSTF, CFIm, and CFIIm complexes, along with 10 additional factors^4,71^ (Fig. 6a; Extended Data Fig. 10a). Although no CPA factor reached significance (p.adj < 0.05) in every tested EN subclass, *CPSF3* and *CPSF4* were consistently downregulated in AD, while *CSTF2T and NUDT21* were upregulated. Several additional factors (*PAPOLA*, *SCAF8*, *PAF1*, *RTF1*, *CDC73*) were also differentially expressed in AD ENs, though their dysregulation was either shared with non-EN cell types or confined to individual EN subclasses. We next asked whether CPA factor fold changes in AD are correlated with 3′-lengthening across EN subclasses (Fig. 6b; Extended Data Fig. 10b). We reasoned that a primary driver of APA should be either upregulated in AD with its expression positively correlated with ΔWUI, or downregulated with negative correlation. *NUDT21* was the leading candidate by this criterion; it was upregulated in AD, and its expression changes were strongly positively correlated with ΔWUI across EN subclasses. Other genes showed weaker or inverse correlations, suggesting weaker association with APA in AD ENs. *NUDT21* upregulation in AD was supported by independent cohorts: Mathys et al.^12^ showed significant upregulation of *NUDT21* in most EN subtypes, while Seattle Alzheimer’s Disease Brain Cell Atlas pseudoprogression analysis revealed a biphasic trajectory of *NUDT21*, downregulation in early stages followed by upregulation in later disease stages^14^.

To further identify regulators of APA in AD EN, we applied weighted gene co-expression network analysis (WGCNA) to CLR-transformed pseudobulk PAS usage profiles, summarized across age-matched (>60) AD and control donors (Methods). We identified six co-regulated modules in the Mount Sinai NIH Neurobiobank (MSSM) cohort, all highly preserved in the Rush Alzheimer’s Disease Center (RADC) cohort (Extended Data Fig. 10c; **Supplementary Table 5**). Modules M2 and M5 represent downregulated intron PAS in AD, whereas M1 comprises 3′ UTR DU-PAS upregulated in AD (Fig. 6c). Similar to our earlier results (Fig. 5g), GO term enrichment analysis showed that synapse regulatory genes were enriched in AD DU-PAS modules. In line with the subclass-level correlation analysis, *NUDT21* expression was strongly correlated with PAS usage in modules M1, M2, and M5, ranking in the top 0.7%, 0.9%, and 0.08%, respectively, across all ~16,000 tested genes. We then expanded the set of candidate APA drivers using a combined rank derived from their correlation with EN subclass-level ΔWUI in AD (Fig. 6b) and with the WGCNA modules (Fig. 6c). *NUDT21* ranked 6th among all candidates under this criterion, and no higher-ranked gene had a known, direct role in APA (Fig. 6d; **Supplementary Table 6**). Other top candidates include a transcription factor (*JUND*)^72^, proteins involved in RNA processing (*HNRNPH2*, *SRSF8*)^73^, and transcription/chromatin regulators (*SUPT20H*, *MLF2*, *MORF4L1*)^74–76^, which may also influence APA despite not being core components of the CPA complex.

Since each component of the CPA complex binds to different PAS-determining motifs^2,4^, we analyzed motif enrichment in up-regulated 3′ UTR PAS and down-regulated intron PAS, further stratified by WGCNA modules. AD down-regulated intron PAS in M2 and M5 modules showed strong depletion of the upstream UGUA motif (p = 9e-5), which is recognized by the CFIm complex (*NUDT21*, *CPSF6*, and *CPSF7*), and enrichment of the downstream G-rich motif (p = 3e-4), targeted by the hnRNP H family including *HNRNPH2*^77^ (Fig. 6e). Other motifs, AAUAAA, U-rich, and GUCU, showed weaker differences (Extended Data Fig. 10d), suggesting that intronic PAS shifts in the M2 and M5 modules are specific to CFIm- and hnRNP H-associated elements rather than reflecting a general change in PAS strength. In contrast, across M1 3′ UTR PAS, the frequency and strength of all PAS-determining motifs were positively correlated with one another, and none showed strong enrichment comparable to that seen at intronic PAS (p = 0.003–0.9; Extended Data Fig. 10e,f).

To further corroborate our observations, we leveraged previously published *in vitro* studies. *NUDT21* targets identified by knockdown followed by RNA-seq^58^ or crosslinking and immunoprecipitation (CLIP)^78^ were significantly enriched among DUGs showing 3′-lengthening in AD excitatory neurons (Fig. 6f). In addition, a previous study using Perturb-seq^22^ showed results consistent with our observations on CPA factor differential expression and APA in AD. For example, *CPSF3* and *CPSF4* were downregulated in AD ENs, and their knockdown in HEK293FT or K562 cells resulted in 3′ UTR lengthening. *NUDT21* and *CPSF6* showed the opposite pattern: 3′ UTR shortening and higher intronic PAS usage upon knockdown, consistent with their upregulation and 3′-lengthening in AD. PAS-level perturbation effects in AD ENs correlated negatively with the effects of *NUDT21*, *CPSF6*, and *CDC73* knockdown, and positively with the effect of *CSTF2* and *PCF11* knockdown, in both K562 and HEK293FT cells (Extended Data Fig. 10g). *NUDT21* and *CPSF6* largely share their targets^22^, consistent with the similar correlation of their knockdown profiles with AD ENs. Together, these convergent observations implicate CFIm dysregulation, and *NUDT21* in particular, in AD-associated APA remodeling. Additional 3′-end processing factors such as *CPSF3* and hnRNP H family members likely contribute as well.

## Discussion

This study identifies mRNA 3′-end selection as a major dimension of cell-type-specific regulation in the human brain and reveals its coordinated remodeling in AD. Increasing AD pathology was associated with reduced intronic cleavage and increased distal PAS usage in excitatory neurons, oligodendrocytes, and immune cells, affecting synaptic and RNA-processing genes in particular. These directional changes were reproduced across independent human datasets and were only partly captured by gene-level differential expression. Moreover, 3′ UTR usage was independently associated with protein changes in synaptic modules^70^. Together, these findings indicate that AD alters not only transcript abundance but also transcript architecture, revealing a layer of molecular pathology that is incompletely captured by conventional gene-level analyses.

A central implication is that gene-level expression and mRNA 3′-end selection capture partly distinct aspects of AD biology. Alternative use of intronic and 3′ UTR PAS can alter coding potential, transcript stability, localization, and translational regulation^2,4^. In our data, differential gene expression and differential PAS usage converged on related synaptic functions but involved largely non-overlapping sets of genes. Reduced intronic PAS usage may favor full-length transcripts, whereas distal 3′ UTR selection may alter post-transcriptional regulation. Because snRNA-seq primarily captures nuclear RNA, the downstream effects of these changes on cytoplasmic RNA and translation will require direct evaluation.

Multiple analyses converged on altered CFIm activity as a candidate regulatory axis for AD-associated 3′-end remodeling. *NUDT21* expression was increased in AD excitatory neurons; its expression tracked 3′-lengthening across neuronal subclasses and PAS-usage modules; AD-downregulated intronic PAS were depleted of the CFIm-recognized UGUA motif; and previously defined *NUDT21* targets were enriched among AD-lengthened transcripts. These findings are directionally consistent with studies showing that reduced *NUDT21*/CFIm25 activity promotes proximal PAS selection and 3′ UTR shortening^58,78^, and with perturbation data for *NUDT21* and *CPSF6*^22^. In previous studies, genetic alteration of *NUDT21* disrupted polyadenylation-site selection and caused cognitive phenotypes in mice and humans^7,79^. Reduced dosage of the other CFIm component *CPSF6* increases intronic polyadenylation and reduces proteins with neuronal function in humans and zebrafish^80^. Together, our results are consistent with increased CFIm-associated activity contributing to distal PAS selection in AD, although they do not establish *NUDT21* as the sole or causal driver. Changes in *CPSF3*, *CPSF4*, and hnRNP H-associated sequence elements further suggest contributions from additional components of the 3′-end processing machinery.

The enrichment of APA remodeling among synaptic genes is notable because synaptic degeneration is closely linked to cognitive impairment in AD^9,10^. An important unresolved question is whether 3′-end remodeling contributes to neuronal dysfunction or instead represents a compensatory response to progressive pathology. Addressing this will require direct perturbation of candidate regulators and individual PAS, together with measurements of cytoplasmic RNA, protein output, and additional brain regions and disease stages. More broadly, our findings establish cell-type-specific mRNA 3′-end remodeling as a reproducible layer of AD molecular pathology that extends beyond changes in gene abundance alone.

## Methods

### Human postmortem brain samples

Sample collection, donor selection, and clinical characterization for the PsychAD cohort have been described previously^11,18^. Briefly, tissue was obtained from three sources, the Mount Sinai NIH Neurobiobank (MSSM), the Rush Alzheimer’s Disease Center (RADC), and the NIMH Human Brain Collection Core (HBCC). For replication analyses, “Mathys” denotes the external snRNA-seq dataset of Mathys et al.^12^, which was generated from the Religious Orders Study and Rush Memory and Aging Project (ROSMAP) cohort; six donors were shared between the PsychAD RADC (n = 152) and the Mathys dataset (n = 406).

### AD diagnostic measurement and differential expression/usage analysis

AD diagnostic measurements, including cognitive, neuropathological, and medical evaluations, were performed according to established procedures^81^, and have been described in detail previously^11,82^. Because individual AD pathological indices are highly correlated, we derived a continuous aggregated pathology metric by extracting the first principal component (DxPC1) from the following measures: Consortium to Establish a Registry for Alzheimer’s Disease (CERAD)^83^, Braak neurofibrillary tangle score (Braak_AD)^84,85^, mean density of neuritic plaques (PlaqueMean), assessment of dementia based on the Clinical Dementia Rating scale^86^, brain region specific Neurofibrillary tangle burden (middle frontal gyrus, and entorhinal cortex), and Neuritic plaque burdens in the middle frontal gyrus, superior temporal gyrus, and entorhinal cortex. Analyses were limited to donors ≥60 years of age without major comorbidities from the PsychAD MSSM brain bank. For the RADC cohort, DxPC1 scores were imputed using a linear model based on CERAD and Braak staging. For all downstream linear models, including differential expression and differential usage tests, DxPC1 was z-scaled across all retained MSSM and RADC donors jointly (denoted DxPC1_z hereafter) to improve the numerical stability of model fitting.

### Polyadenylation site (PAS) annotation and quantification

PAS annotation and quantification procedures were adapted from the previously published SCAPTURE pipeline^20^, with in-house modifications for the PsychAD dataset: 1) in the mergesample module, we used the full peak region with a 30% overlap threshold when merging peaks from exons, introns, and 3′ downstream regions within the same sample, to maintain consistency with the downstream peak merging process, and 2) for UMI quantification, only uniquely mapped reads (mapping quality = 255) were counted using umi_tools.

For peak calling, 5,000 cell barcodes were randomly selected from each combination of age group (2–11yr, 12–19yr, 20–39yr, 40–59yr, 60yr+) and six major brain cell types (IN, EN, Oligo, OPC, Astro, and Immune). The neonatal group (< 2yr) and the Endo, Mural cell types were excluded owing to insufficient available nuclei. Sequencing reads from selected cells, collapsed by unique molecular identifier (UMI) to remove PCR duplicates, were subjected to peak calling on Ensembl transcripts (v104, GRCh38), using the SCAPTURE *callpeak* module. For each called peak, we defined a test interval spanning 50 bp upstream to 25 bp downstream of its 3′-end, measured along the peak blocks so that spliced peaks were handled correctly. A peak was considered to match an annotation when this interval overlapped a record of that annotation on the same strand by at least 1 bp. Called PAS peaks were retained if they met one of the following criteria: a) matched GENCODE (v38) polyA sites (polya_site records in gencode.v38.polyAs.gtf), b) matched at least two of the following databases, or c) matched one of the following databases and passed DeepPASS prediction provided by SCAPTURE. The following four databases were used for filtering: i) PolyA_DB3 (downloaded 2024/Nov/29), ii) PolyA-seq (downloaded 2024/Nov/29), iii) PolyASite2.0 (downloaded 2024/Nov/27), and iv) PolyASite3.0 (downloaded 2024/Nov/27). For PolyASite3.0, only PAS expressed above 0.1 RPM in blood or brain tissues were used.

The resulting PAS annotations contained two levels of redundancy arising from overlapping genes and independent identification across cell types and age groups. To assign each PAS to a single primary gene, we applied the following prioritization criteria in order: 1) higher gene-level expression in the snRNA-seq data, 2) higher expression in long-read sequencing (MAS-seq), 3) transcriptomic location of the PAS within the gene (prioritized as: 3′ UTR > CDS > non-coding exon > intron > 3′ downstream > 5′ UTR), and 4) longer gene length. All PAS-gene mapping information was retained for downstream analyses, including PAS usage testing. To merge redundant PAS identified independently across cell types and age groups, we identified groups of overlapping PAS using bedtools^87^ (v2.27.1) intersect (requiring >30% overlap with at least one other PAS), then merged each group using bedtools merge.

Transcriptomic regions were reassigned to PAS after deduplication, to resolve conflicting annotations within merged PAS. For higher-level regional assignment, we used the original classifications from SCAPTURE, with the following priority: 3′ UTR > CDS > non-coding exon > intron > 3′ downstream > 5′ UTR. For finer 3′ UTR classifications, we defined the provisional 3′ UTR as the interval between the outermost 3′-ends of CDS and transcripts per gene. PAS with 3′-ends falling within the first or second half of the provisional 3′ UTR were classified as proximal or distal 3′ UTR, respectively, while those upstream were classified as upstream 3′ UTR. For genes without annotated CDS but containing 3′ UTR PAS according to SCAPTURE (48 PAS), 3′ UTR PAS were reclassified as non-coding exons if overlapping an exon, or as intronic otherwise.

Finally, the SCAPTURE *PASquant* module was applied to quantify UMI counts for each PAS in individual cell barcodes. For pseudobulk-level analyses, nuclei were aggregated by library and cell type. PAS were then filtered within each tissue source (MSSM, RADC, HBCC) and the combined dataset (FULL) to retain features expressed at >1.0 CPM in at least 20% of pseudobulk libraries.

### Variance partition analysis of gene and PAS expression

To identify contributions from biological and technical factors on PAS variation, we applied *variancePartition* (1.36.3) to gene- and PAS-level expression^32,33^. Pseudobulk read counts were generated by aggregating reads across nuclei within each library and class-level cell type, then assays (cell types) were stacked using the StackedAssay() function of Dreamlet (v1.4.1)^37^. PAS-level read counts are sparser and may be more susceptible to technical biases due to differing GC content across transcriptomic regions (i.e., lower GC content in 3′ UTRs)^88^, thus we included GC contents and genomic region related technical covariates in addition to previously used variables^11^. We therefore used the following regression formula:

$$\text{Gene or PAS expression}\sim \left(1|\text{stackedAssay}\right)+\left(1|\text{Sex}\right)+\left(1|\text{Channel}\right)+\left(1|\text{SubID}\right)+\left(1|\text{Brain\_bank}\right)+\left(1|\text{MajorDisease}\right)+\text{scale}\left(\text{Age}\right)+\text{scale}\left(\text{PMI}\right)+\text{log}\left(\text{n\_genes}\right)+\text{percent\_mito}+\text{ribo\_genes}+\text{scale}\left(\text{GC\_NC\_20\_39}\right)+\text{scale}\left(\text{MEDIAN\_5PRIME\_TO\_3PRIME\_BIAS}\right)$$

For downstream analyses, biological factors (cell type (stackedAssay), age, sex, donor ID (SubID), major disease) and pre-sequencing technical factors (technical replicates (Channel), brain bank (tissue source), post mortem interval (PMI)) were retained as separate components. Variances from post-sequencing technical factors (n_genes, percent_mito, ribo_genes, GC_NC_20_39, and 5′ to 3′ ratios) were summed and renamed as ‘SeqQC scores’. MajorDisease is a categorical variable indicating disease status: Alzheimer’s disease (AD), schizophrenia (SCZ), bipolar disorder (BD), mild cognitive impairment (MCI), or control. For the gene or PAS filtering during the voom process, read count threshold was lowered by setting parameter *min.prop* to 0 in Dreamlet function *processAssays*, to retain maximum number of genes and PAS.

### Differential expression analysis of gene and PAS using Dreamlet

To systematically compare gene and PAS expression across cell types or disease status, we applied the Dreamlet^37^ framework, which adopts pseudobulk approaches for the cell-type-specific differential expression analysis while accounting for random effects by linear mixed models. We followed our previous workflow^11^ for the major part of the differential expression analysis pipeline, with a few modifications. First, min.prop parameters of processAssays() and dreamletCompareCluster() functions were set to 0.2, to lower the read count cutoff owing to the higher sparsity of PAS counts. Secondly, we modified the regression formulae, which will be described separately for the cell-type comparative analysis, AD versus control analysis, and WGCNA analysis.

### Compositional usage analysis of PAS by Crumblr and Dream

Analysis of count ratios (e.g., fraction of transcript expression in total gene expression) requires sophisticated methods because these proportions are non-normal and inherently interdependent—an increase in one fraction necessitates decreases in others. To account for this, we applied the centered log-ratio (CLR) transform by Crumblr (0.99.19), enabling differential usage analysis using linear model-based tools such as Dream. This Crumblr-Dream framework has been successfully applied to measure cell type composition changes in brains under different conditions^11,38,40,89^, and we repurposed it here to assess PAS usage changes.

Briefly, for each gene containing at least one PAS that passed the count filtering for differential expression analysis (performed by processAssay() or dreamletCompareCluster() in Dreamlet), we collected the UMI counts of PAS within that gene at the pseudobulk level. To filter out low-expressed PAS within each cell type, only PAS with ≥ 5 UMI counts in more than 20% of samples were retained. For the subclass-level analyses shown in Fig. 5 and Extended Data Fig. 10, this count filter was applied at the class level (e.g., the EN level for all EN subclasses) so that the same set of PAS was used across subclass comparisons. For all-PAS analyses, every PAS passing the count filter was included, whereas for 3′ UTR PAS analyses only PAS annotated as 3′ UTR or 3′ downstream were retained.

For each gene, PAS counts were transformed with Crumblr, and the transformed values were concatenated across all tested genes and subjected to differential expression analysis using dream() in variancePartition^33^. To obtain interpretable usage estimates, we back-transformed CLR coefficients to the original compositional ratios with the inverse CLR transformation, clrInv(). Specifically, for the PAS of each gene we computed the average, test, and control usages by applying clrInv() to the vectors of AveExpr, AveExpr+logFC/2, AveExpr-logFC/2 from the same gene, respectively. Finally, we took the inner product of the PAS usage vectors with a positional weight vector to obtain the weighted usage index (WUI) per test and control, as illustrated in Fig. 3c. ΔWUI was defined as the difference between the test and control WUI values. For the ΔWUI along DxPC1 across the subclass-level cell types in Fig. 6 and Extended Data Fig. 10, low-expressed PAS filtering before Crumblr was done at the class-level cell types and applied to all subsequent subclass types, to avoid comparing the WUI values obtained from different sets of PAS per single gene.

### Class-level cell type comparative analysis

To systematically identify cell-type-specific gene and PAS expression, we performed cross-cell-type differential expression analysis by dreamletCompareCluster() from Dreamlet, which compares pseudobulk count tables of one cell type against all other cell types. Pseudobulk counts were aggregated at the channel (library) level by combining nuclei within each library and cell type combination. For PAS usage comparisons, we created a modified version of dreamletCompareCluster()(withvoomCompareCluster.R) that returns the same pseudobulk table structure as dreamletCompareCluster() and performed Crumblr transformation as previously described. To minimize biological variations other than cell type differences, only control samples from donors aged 20 to 60 years were included in the analysis. The regression formula used was:

Expression or usage~0+cell type.

For each class-level cell type, WUI was calculated by first obtaining the total read count of each PAS from the PAS-wise sum of pseudobulk counts across samples within that cell type. WUI differences from the gene-wise mean were then subjected to K-means clustering, performed using the Python scikit-learn package (v1.4.0).

### Differential expression and usage analysis for AD

For binary AD versus control analysis, we used AD and control donor definition in our previous study^11^. We tested differential expression and usage along binary AD versus control or DxPC1 across four molecular layers with Dreamlet or Crumblr-Dream, as previously described: 1) gene expression, 2) PAS expression, 3) PAS usage, and 4) regional usage (summing PAS usages within transcriptomic regions such as CDS and 3′ UTR). All tests were performed at the donor level (SubID). The following formulae were used:

$$\text{Expression}\sim \left\{\left(0+\text{AD}\right)\text{or DxPC1\_z}\right\}+\left(1|\text{Brain\_bank}\right)+\text{scale}\left(\text{Age}\right)+\text{Sex}+\text{scale}\left(\text{PMI}\right)+\text{log}\left(\text{n\_genes}\right)+\text{percent\_mito}+\text{ribo\_genes}+\left(1|\text{SubID}\right)+\text{scale}\left(\text{GC\_NC\_20\_39}\right)+\text{scale}\left(\text{MEDIAN\_5PRIME\_TO\_3PRIME\_BIAS}\right)$$

$$\text{Usage}\sim \left\{\left(0+\text{AD}\right)\text{or DxPC1\_z}\right\}+\left(1|\text{Brain\_bank}\right)+\text{scale}\left(\text{Age}\right)+\text{Sex}+\text{scale}\left(\text{PMI}\right)+\text{percent\_mito}+\text{ribo\_genes}+\left(1|\text{SubID}\right)+\text{PCT\_INTRONIC\_BASES}+\text{scale}\left(\text{GC\_NC\_20\_39}\right)+\text{scale}\left(\text{MEDIAN\_5PRIME\_TO\_3PRIME\_BIAS}\right)$$

Note that the expression model includes log(n_genes) to account for library size and quality, while the usage model includes PCT_INTRONIC_BASES to account for global transcriptomic regional biases more directly. For both PAS expression and usage differential testing, Benjamini-Hochberg multiple-testing corrections were performed across all PAS/genes within each cell type.

For the AD versus control analysis of Mathys dataset^12^, raw snRNA-seq data were first preprocessed for sample quality control and cell taxonomy assignment with an in-house pipeline as previously described^11^. Of 427 donors, 406 passed quality control, and nuclei were assigned to seven class-level cell types, with Endo and Mural merged into a single Vascular class. Differential expression and usage along binary AD diagnosis, CERAD, and Braak were assessed by the same methods, but with simplified regression formulae. A single model including all three diagnostic variables was used for the voom step, and each variable was tested separately thereafter:

$$\text{Voom}:\sim \text{Sex}+\text{scale}\left(\text{Age}\right)+\text{scale}\left(\text{PMI}\right)+\left(1|\text{BRAAK}\right)+\left(1|\text{CERAD}\right)+\left(1|\text{AD}\right)+\text{log}\left(\text{n\_genes}\right)+\text{percent\_mito}+\text{mito\_ribo}+\text{ribo\_genes}$$

$$\text{Expression or usage}:\sim \left\{\text{AD or BRAAK or CERAD}\right\}+\text{Sex}+\text{scale}\left(\text{Age}\right)+\text{scale}\left(\text{PMI}\right)+\text{log}\left(\text{n\_genes}\right)+\text{percent\_mito}+\text{mito\_ribo}+\text{ribo\_genes}$$

### Cell-type-specific open chromatin region analysis

Single-nucleus multiome (snRNA-seq + snATAC-seq) profiles were generated from postmortem human brain tissue spanning prefrontal cortex, superior temporal gyrus, and entorhinal cortex from 247 PsychAD donors. Cell taxonomy was derived by integrating RNA and ATAC modalities using a weighted nearest neighbor approach^90^, and was confirmed by canonical marker genes and label transfer to the PsychAD snRNA-seq atlas using ScanVI/scVI^91,92^. Chromatin accessibility peaks were called within each major cell class using ArchR (v1.0.2)^93^ with MACS2 (v2.2.7.1)^94^ on aggregated fragments per class. Major cell-type–enriched differentially accessible peaks were identified using ArchR getMarkerFeatures, conditioning on TSSEnrichment and log10(nFrags). Peaks were defined as cell-type enriched if they met false discovery rate (FDR) ≤ 0.01 and log2FC ≥ 1. The resulting peak sets were used as a reference resource for downstream analyses and cross-modality interpretation.

### Brain myeloid cell subtype comparative analysis

Due to the limited number of nuclei in individual myeloid cell subtypes, we included all nuclei from donors aged 20 years or older in this analysis. For differential gene expression, ~1,000 cells per cell type were subsampled and tested using Wilcoxon’s rank-sum test (scanpy v1.11.5). For differential PAS expression, ~3,000 cells per cell type were subsampled and tested similarly. For differential PAS usage, we generated 10 pseudo-replicate samples per cell type, each aggregating approximately 1,000 nuclei. These were compared against 10 pseudo-replicates from all other myeloid cell types combined, with each combined sample pooling approximately 1,000 nuclei from each of the three remaining cell types for a total of approximately 3,000 nuclei. Differential usage testing was performed using the Crumblr-Dream framework as described in previous sections.

### Disease genetic heritability analysis by MAGMA and LDSC

Association between gene sets and disease-associated genetic variants was tested by Multi-marker Analysis of GenoMic Annotation (MAGMA, v1.10)^29,30^. SNPs were mapped to genes using a standard window of 35 kb upstream and 10 kb downstream of the gene body for autosomal protein-coding genes. To test the enrichment of disease heritability in genes (5′- to 3′-end), PAS regions (−700 bp to +300 bp from 3′-end cleavage sites), and open chromatin regions (OCRs), we performed Linkage Disequilibrium SCore regression (LDSC, v.1.0.0) using the 1000 Genomes Phase 3 European ancestry reference panel. We used the following genome-wide association study (GWAS) summary stats from studies for MAGMA and LDSC: AD^52^, attention-deficit/hyperactivity disorder^95^, amyotrophic lateral sclerosis^96^, autism spectrum disorder^97^, bipolar disorder^98^, major depressive disorder^99^, migraine^100^, multiple sclerosis^101^, Parkinson’s disease^102^, Schizophrenia^103^, and Stroke^104^. Both MAGMA and LDSC analyses excluded the major histocompatibility complex (MHC) region. To enable comparison across modalities of different coverage, we utilized the regression coefficient (normalized tau), and its P-value (hypothesis, tau > 0). The normalized tau measures the average per-SNP contribution of the annotation to heritability.

### MAS-Seq library preparation and sequencing

10x Genomics Multiome cDNA concentrations and length were determined by Qubit DNA HS assay kit (Invitrogen, cat# Q33230) and Agilent Bioanalyzer DNA HS kits (Agilent, cat# 5067–4626), respectively. Using the MAS-Seq for 10× 3′ concatenation kit (PacBio, cat# 102-407-900), the MAS capture beads kit (PacBio, cat# 102-428-400) and the MAS-Seq for 10x Single Cell 3′ kit (PacBio, cat# 102-659-600), 15 ng of each sample was used to generate MAS-Seq libraries, according to manufacturer’s instructions. Resulting cDNA concentrations were measured by Qubit DNA HS assay. 50 ng of cDNA per sample was split into 16 PCR reactions with 16 asymmetric primer adapter pairs. After 9 cycles of PCR, the 16 reactions were pooled and cleaned with 1.5x SMRTbell cleanup beads (PacBio, cat# 102-158-300). 10 μg of this MAS amplicon pool was then used for MAS array formation. The cDNA segments were ligated into a concatenated 16-mer array, and MAS adapters were added to the ends of the arrays. Nicked and damaged DNA sites in the array were repaired, and the MAS arrays cleaned with 1.2x SMRTbell clean-up beads. Incomplete or damaged MAS arrays were digested in a final nuclease treatment step. The final libraries were cleaned using 1.2x SMRTbell cleanup beads and quantified by Qubit dsDNA HS assay and on an Agilent Femto Pulse using the Genomic DNA 165 kb kit (Agilent, cat# FP-1002–0275). Libraries were separately annealed to the MAS sequencing primer and bound to the Sequel II polymerase for sequencing on individual 8M SMRTcells on the Sequel II system. CCS reads were generated from the raw data filtered for only CCS reads with high accuracy (>Q20), at least 3 polymerase passes, and acceptable barcode quality scores as HiFi reads. PacBio’s standard workflow for MAS-Seq libraries was run in SMRTLink, including segmentation of the 16-mers using skera, single-cell demux, and transcript classification using SQANTI3^105^.

### Nanopore library preparation and sequencing

Full-length cDNA sequencing libraries were prepared using 5–10 ng input of the unfragmented 10x Genomics cDNA amplicons per sample following the Oxford Nanopore Technologies protocol ‘Single-cell transcriptomics sequencing from 3′ cDNA prepared with 10x Genomics using SQK-LSK114’ (https://nanoporetech.com/document/single-cell-transcriptomics-with-cdna-prepared-using-10x#post-pull-down-pcr). Libraries were sequenced on a PromethION 24 sequencer using R10.4.1 flow cells (Oxford Nanopore Technologies). Raw reads were basecalled using Dorado v.0.7.2 with the super accuracy (SUP) v.5.0.0 model. Basecalled FASTQs were analyzed using the Oxford Nanopore Technologies EPI2ME^™^ wf-single-cell v2.1.0 pipeline (https://github.com/epi2me-labs/wf-single-cell). In brief, the workflow performs adapter identification, read preprocessing, mapping to a reference, gene and transcript assignment, cell barcode and UMI extraction and correction, generation of gene- and transcript-level UMI count matrices, and library saturation calculation.

### Weighted Gene Co-expression Network Analysis (WGCNA) of PAS usages in excitatory neurons

To identify modules of coordinately regulated PAS and associate them with disease traits, we performed WGCNA^106^ on PAS usage data from Crumblr-Dream framework. First, we extracted CLR-transformed PAS usage matrices (E values from Crumblr objects) generated for MSSM/RADC ENs from donors used in DxPC1-based AD versus control analysis. To regress out technical covariates from PAS usages, the following linear model was fitted per each PAS for its usages, using Dream.


$$\text{PAS usage}\sim \text{DxPC1\_z}+\text{mod\_BRAAK\_AD}+\text{scale}\left(\text{Age}\right)+\text{Sex}+\text{scale}\left(\text{PMI}\right)+\text{percent\_mito}+\text{ribo\_genes}+\text{scale}\left(\text{MEDIAN\_5PRIME\_TO\_3PRIME\_BIAS}\right)+\text{mod\_CERAD}\left(\text{only MSSM}\right)+\text{scale}\left(\text{mod\_Plq\_Mn}\right)\left(\text{only MSSM}\right)$$

Then we extracted regression coefficients from models, to subtract effects from PMI, percent_mito, ribo_genes, MEDIAN_5PRIME_TO_3PRIME_BIAS. Only PAS from protein-coding genes were retained for analysis. WGCNA module assignment was done by blockwiseModules() function, using a signed network with soft-thresholding power of 8. Modules with distance less than 0.25 were merged. To rank potential regulator genes by their co-expression with PAS modules, gene expression values were taken as voom-normalized log_2_CPM (E values) from the dreamlet-processed pseudobulk of the same cell type and donors, and corrected for the same four technical covariates using coefficients from a matching Dream fit. Pearson’s correlation between the corrected gene expression and each module eigengene (the first principal component of the PAS usage in that module) was then used to prioritize candidate regulators. For the gene functional annotation of potential regulators in Fig. 6d, following databases are used: Cantarella et al.^107^ for RNA binding proteins; Choi et al.^108^ for early life cycle RNA binding proteins; Lambert et al.^72^ for transcription factors. Transcription and chromatin regulators are marked manually.

### Pathway enrichment analysis

MSigDB (v7.0)^31^ and Gene Ontology (GO) enrichment analyses were performed using clusterProfiler (v4.14.4)^109^. Similar (Jaccard index > 0.5) MSigDB terms were merged and only the largest term was retained. GO annotations were obtained from the R package org.Hs.eg.db (v3.20.0), and redundant GO terms were reduced using rrvgo (v1.18.0)^110^.

### Statistical testing and data visualization

Annotation terms were abbreviated as follows: regulation (reg.), positive (pos.), negative (neg.), modification (mod.), and conjugation (conj.) All violin plots were generated using the Python package seaborn (v0.13.2) with the cut=0 option to restrict kernel density estimation to the observed data range. All adjusted p-values (p.adj) were derived from p-values using Benjamini-Hochberg procedure, and q-values were calculated using the qvalue package (v2.38.0) in R. A gene or PAS was identified as differentially expressed if p.adj < 0.05. A PAS was identified as differentially used if p.adj < 0.05 and had an average usage > 0.05 across all tested samples. A gene was defined as DUG if any PAS was differentially used.

### Motif analysis

Strand-specific genomic sequences around each PAS 3′-end were extracted with bedtools (v2.27.1) getfasta. Motifs were searched with the Python regex module (v2.5.140), allowing overlapping matches, using the following regular expressions: ‘(?!AAAAAA)(AAUAAA){s<=1}’ for AAUAAA and its 1 nt variants, ‘UGUA’ for UGUA, ‘U(UUUUU){s<=1}’ for U-rich, ‘GUGU|UGUG|GUCU|UGUC|CUGU|UCUG’ for GU/CU-rich, and ‘G(GGGGG){s<=1}’ for G-rich. To realign motif positions to the polyadenylation signal, we (1) searched for the 3′-most occurrence of AAUAAA and of its 1 nt variants within −80 to 0 bp relative to the annotated PAS 3′-end, and (2) among PAS carrying such a signal, anchored motif positions to the exact AAUAAA when present, or to the 1 nt variant when no exact AAUAAA was found. P-values for motif under- or over-representation were calculated by Mann-Whitney U test on per-PAS motif counts between groups, where the count for each PAS was defined as the number of motif matches within the visualized position window, extended at both ends by the motif length minus one nucleotide. Visual guides of expected motif positions (vertical lines in Fig. 6e and Extended Data Fig. 10d,e) were adapted from a previous study^26^.

### Proteome data analysis

AD-control protein fold changes were taken from Johnson et al^70^, keeping one protein group per gene symbol in the order of (1) higher number of quantified peptides, (2) SwissProt over trEMBL, (3) higher number of identified peptides, and (4) higher number of identified peptide-spectrum matches. Per class-level cell type, protein fold changes were paired with differential mRNA expression or 3′ UTR usage fold changes along DxPC1_z, by gene symbols. Lastly, a linear model was fitted on protein fold changes from Johnson et al. and excitatory neuron transcriptomic changes in PsychAD, as logFC_prot ~ logFC_mRNA + logFC_3UTR.

### NUDT21 knock down, PAR-CLIP, and cleavage and polyadenylation factor perturb-seq data reanalysis

To leverage published perturbation and RNA-binding data for NUDT21 and other cleavage and polyadenylation (CPA) factors, we reanalyzed siRNA knockdown of NUDT21 in HeLa cells^58^, photoactivatable ribonucleoside-enhanced crosslinking and immunoprecipitation (PAR-CLIP) of NUDT21 in HEK293 cells^78^, and Perturb-seq targeting multiple CPA factors in K562 and HEK293FT cells^22^. For the knockdown, the reported list of genes with altered 3′ UTR length in the original study (maximum FDR 0.013, ~1400 genes) was compared with our gene-level DxPC1 DUGs. For PAR-CLIP, per-base density wig files were converted to BED with wig2bed (BEDOPS) and summed over contiguous blocks with bedtools merge. Blocks were lifted over from hg19 to hg38 and intersected with Ensembl v104 transcripts on the matching strand, requiring full containment on the transcript. Density was summed per transcript, and the highest-scoring transcript defined each gene’s binding score. Genes scoring above 3,000 in both replicates were called NUDT21 targets. This empirical threshold yielded 2,324 target genes, between the number of targets reported for the siRNA knockdown (~1,400) and for Perturb-seq (~3,000). Enrichment among DUGs was tested by one-sided Fisher’s exact test, using the union of tested DUGs and CLIP targets as the universe.

For Perturb-seq, Seurat objects from GSE269596 were reprocessed with PASTA, an APA analysis pipeline published together with Perturb-seq results. Differential APA was tested by CalcPolyAResiduals() and FindDifferentialPolyA() functions of PASTA. Perturb-seq PAS were matched to ours by nearest 3′ end on the same strand within 100 bp. To avoid artifacts from differing PAS definitions between studies, we compared changes in usage percentage points rather than fold changes. For each pair of a Perturb-seq dataset and a PsychAD cell type, we computed Spearman’s *ρ* across matched PAS, requiring at least 20 matched PAS per pair. Two-sided p-values came from 20,000 permutations shuffling PAS in PsychAD results within transcriptomic subregion strata (5′ UTR, CDS, intron, upstream 3′ UTR, proximal 3′ UTR, distal 3′ UTR, and 3′ downstream).

### Polyadenylation analysis on purified microglia datasets

Two datasets of purified human microglia independent of PsychAD were analyzed, a long-read Iso-seq dataset^56^ and a short-read transcript-level dataset^43^, with all donors of the former also present in the latter. For both datasets, we defined windows spanning 100 bp on either side of each PAS 3′-end, and assigned a long read or a transcript to a PAS when its 3′-end fell within that window. For the long-read data, full-length non-concatemer reads were aligned to GRCh38 with minimap2 using the spliced high-quality preset, and read 3′-ends were counted per PAS 3′-end window with featureCounts in stranded, long-read mode. PAS usage was defined as the proportion of reads assigned to each PAS within a gene and compared between AD and control donors by two-sided Mann-Whitney U test.

Two complementary analyses were performed on the short-read data. First, transcript-level counts were aggregated per PAS, assigning transcripts to the closest 3′-end PAS when a transcript 3′-end fell in more than one PAS 3′-end window. Donors aged 45 years or younger were excluded, and genes with more than one expressed PAS were retained. Aggregated PAS counts were tested for differential expression by Dream, and for differential usage by Crumblr-Dream. Both tests compared AD with control using the CERAD-based diagnosis and the covariate model (including age) of the original study^43^. Second, DaPars^57,58^ was applied to aligned bam files, using 3′ UTR annotations extracted from Ensembl v104 reduced to one representative transcript per gene.

## Extended Data

**Extended Data Figure 1.**
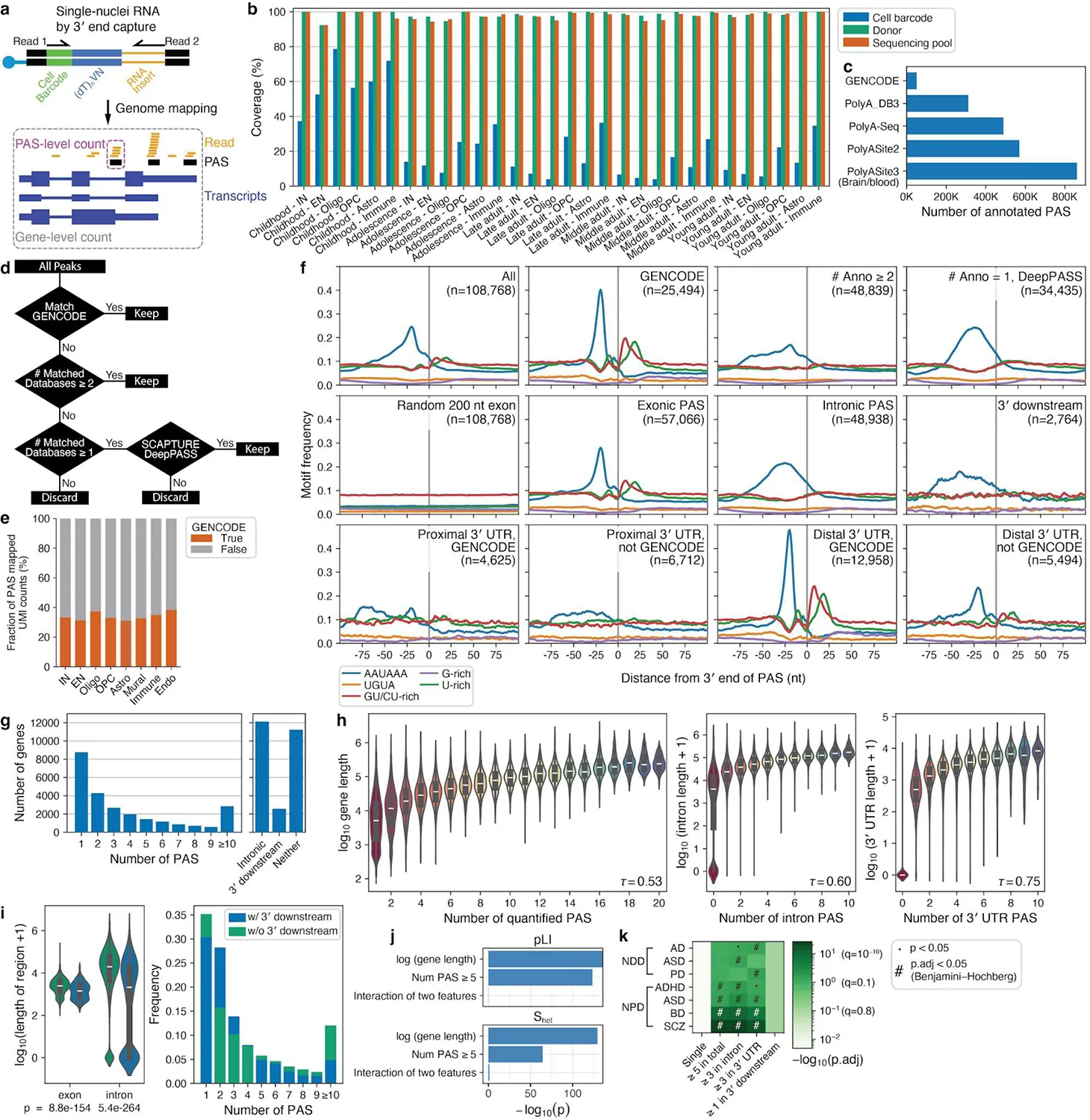
PAS annotation methods and additional characterization **a**, Schematic of gene-level and PAS-level expression measurement from 3′-end capturing snRNA-seq. **b**, Coverage of nuclei, brain donors, and sequencing pools captured by 5,000 subsampled nuclei from each age × cell type group. **c**, Number of annotated PAS in each database used in this study. For PolyASite3, only PAS with RPM > 0.1 in brain or blood tissues were included (subset of ~1.75 million total annotated sites). **d**, Decision tree of filtering PAS using reference databases and deep-learning-based prediction (SCAPTURE DeepPASS). **e**, Fraction of PAS-mapped UMIs by GENCODE annotation status across cell types. **f**, Positional frequency of polyadenylation-associated sequence motifs around the PAS 3′-end. **g**, Histogram of PAS per gene (left) and number of genes with intronic or 3′ downstream PAS (right). **h**, Distributions of gene length (left), total intron length per gene (middle), and 3′ UTR length, by the number of PAS. Per each gene, the longest basic GENCODE transcript was selected for gene, intron, and 3′ UTR length calculation. Kendall’s *τ* correlation coefficient is shown in each panel. **i**, (left) Exonic and intronic region length distribution of genes with (blue) or without (green) 3′ downstream PAS. P-values are derived from the Wilcoxon rank-sum test between two groups. (right) Distribution of the number of PAS for genes with (blue) or without (green) 3′ downstream PAS. **j**, ANOVA p-values from linear models testing gene length, PAS count (≥5), and their interaction as predictors of evolutionary constraint scores (pLI and S_het_). **k**, Conditional MAGMA analysis results, similar to Fig. 1g but adjusted for cell-type-specific gene-level expression.

**Extended Data Figure 2.**
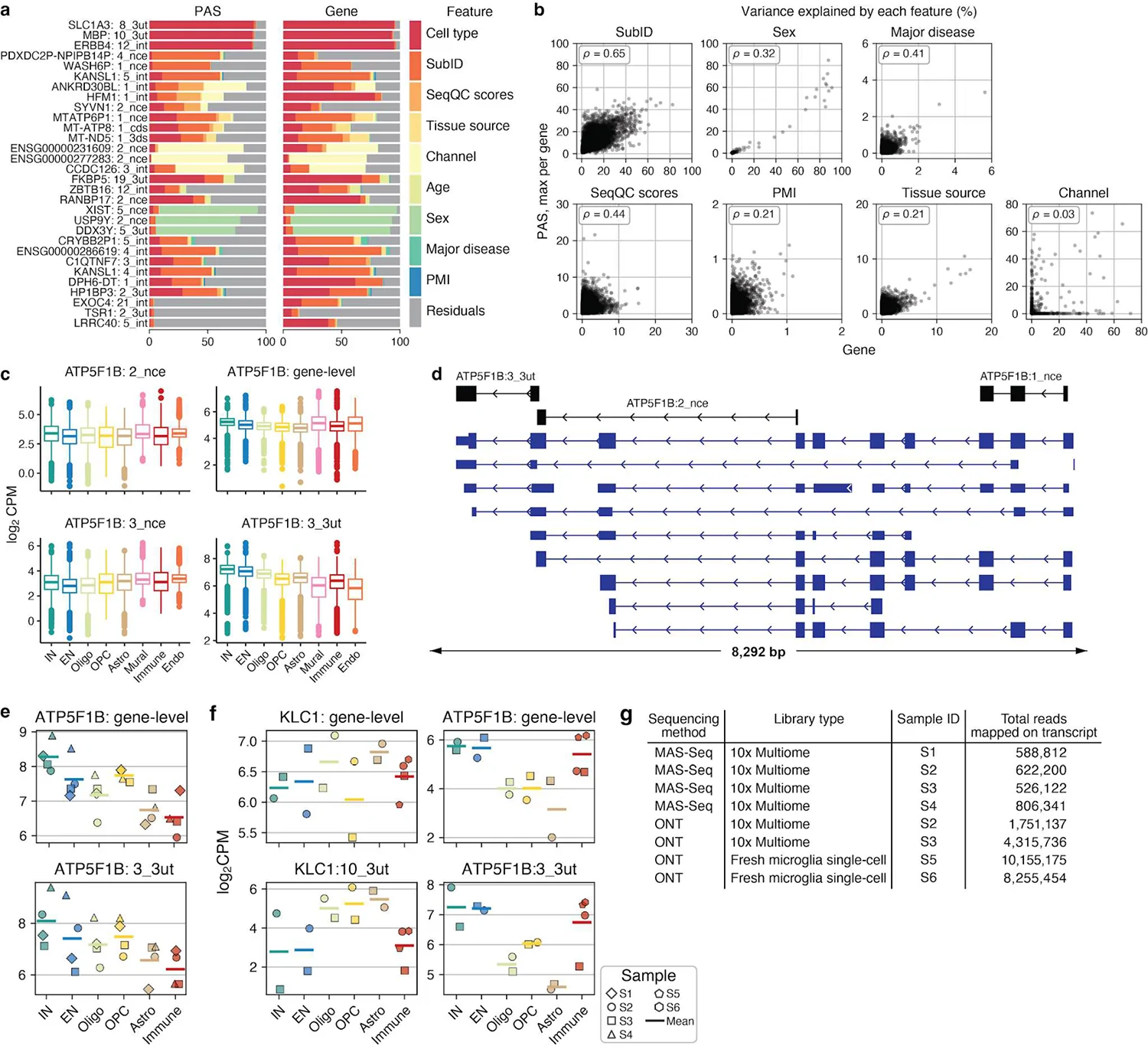
Variance partition of gene and PAS expression, and long-read validation of cell-type variable PAS **a**, For each covariate, three genes with the highest PAS-level variance were selected; the PAS with maximum variance explained is shown for each gene (left), alongside corresponding gene-level values (right). **b**, PAS and gene variances explained by each feature, except cell type and age, which are shown in Fig. 2b. **c**, *ATP5F1B* expression at gene- and PAS-level. log_2_CPM values in this and following panels are normalized per replicate and cell type. **d**, *ATP5F1B* transcript annotation in Ensembl (blue, selected) and PAS annotation in this study (black). **e**, Long-read RNA-seq results for *ATP5F1B*, by Multiplexed Arrays Sequencing. **f**, Long-read RNA-seq validation of *KLC1* and *ATP5F1B* by Nanopore sequencing. Sample markers are shared in **e** and **f**. **g**, Number of reads mapped to transcript, per each long-read library.

**Extended Data Figure 3.**
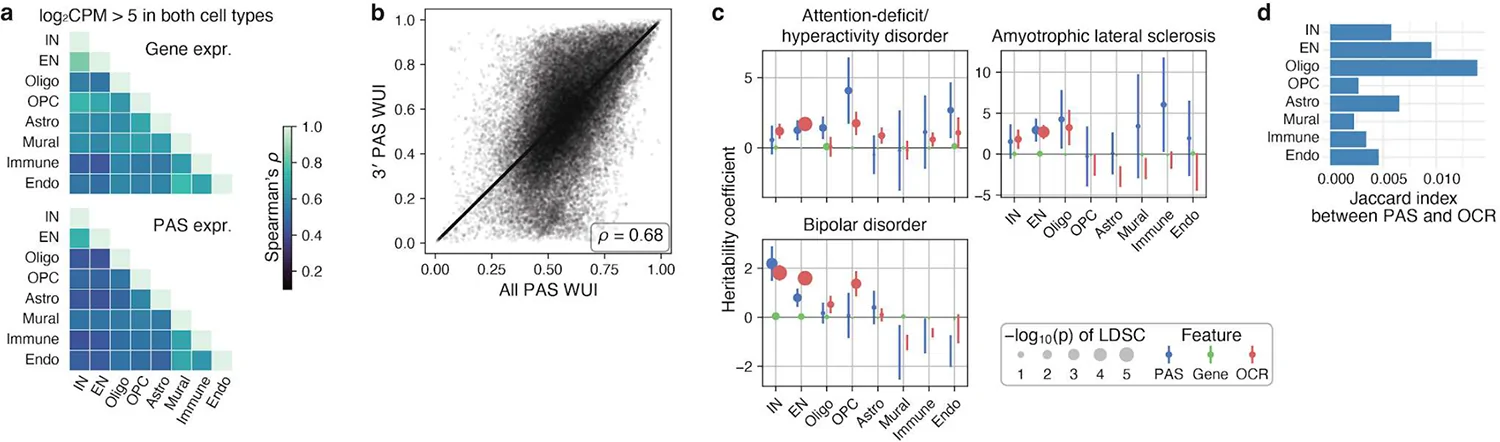
Additional analyses on alternative polyadenylation across class-level cell taxa **a**, Correlation of gene- (upper) and PAS- (lower) level expression (log_2_CPM) between class-level cell types. Unlike Fig. 3b, only genes with log_2_CPM > 5 in both cell types were included. **b**, Scatter plot showing WUI values, derived from all PAS and 3′ UTR PAS of the corresponding gene. Only genes with more than one 3′ UTR PAS were included in the analysis. **c**, Linkage Disequilibrium Score Regression (LDSC) analysis, same as Fig. 3g. **d**, Jaccard indices showing the overlapped portions between cell-type-specific PAS and OCRs, used in LDSC analysis of Fig. 3g and panel **c** of this figure.

**Extended Data Figure 4.**
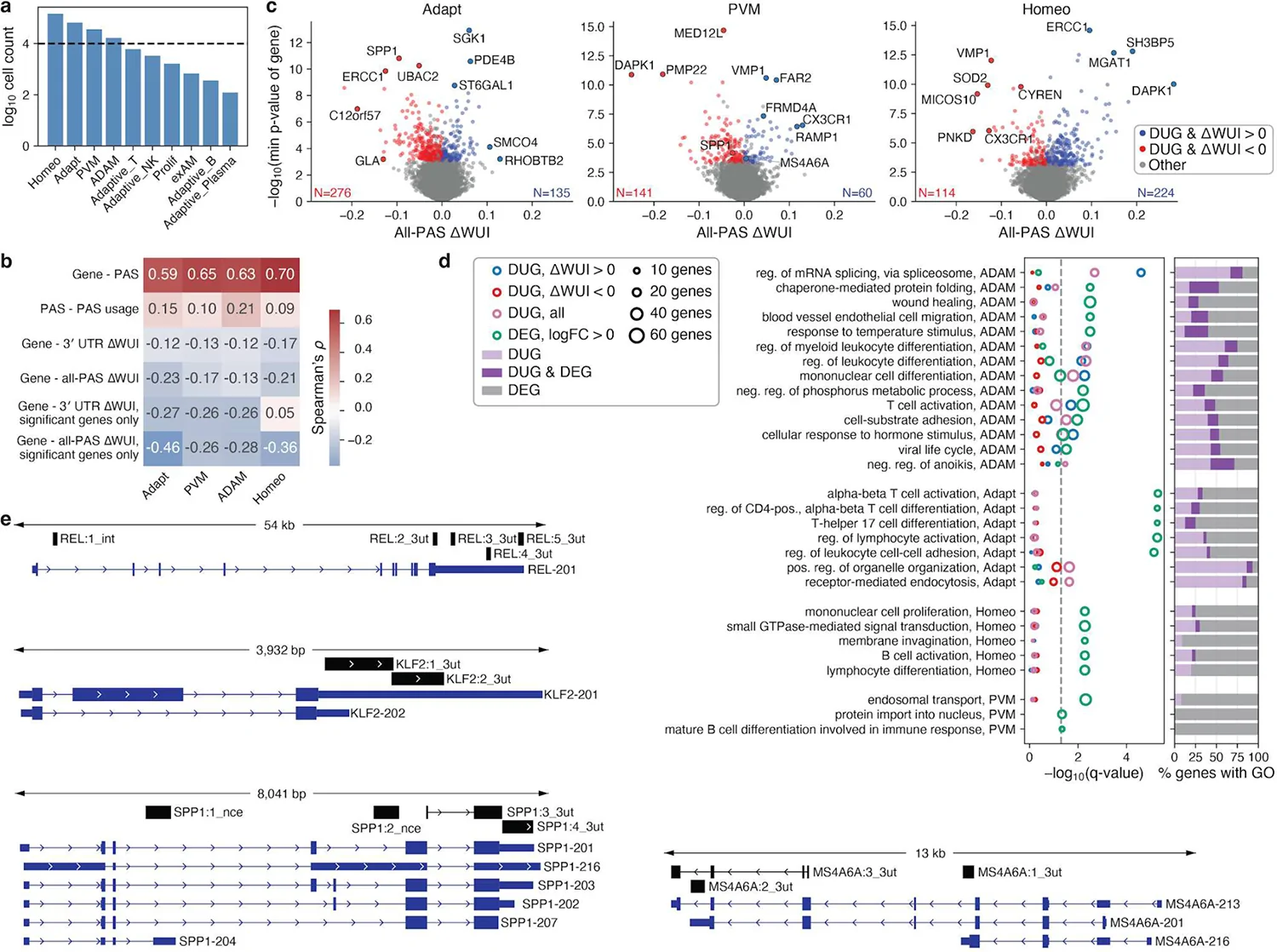
Additional analyses of alternative polyadenylation across brain immune cell subtypes **a**, Number of cells per immune subtype in the PsychAD. Cells from donors under 20 were discarded from this and all other immune subtype analyses. **b**, Spearman’s *ρ*, defined as in Fig. 3a but across PVM and microglial states. **c**, Volcano plot, same as Fig. 4c. **d**, GO: biological process terms enriched in upregulated DEGs or all-PAS DUGs of each cell type. **e**, Transcripts and PAS of *REL*, *KLF2*, *SPP1*, and *MS4A6A* genes. Only selected transcripts of Ensembl reference v104 are shown.

**Extended Data Figure 5.**
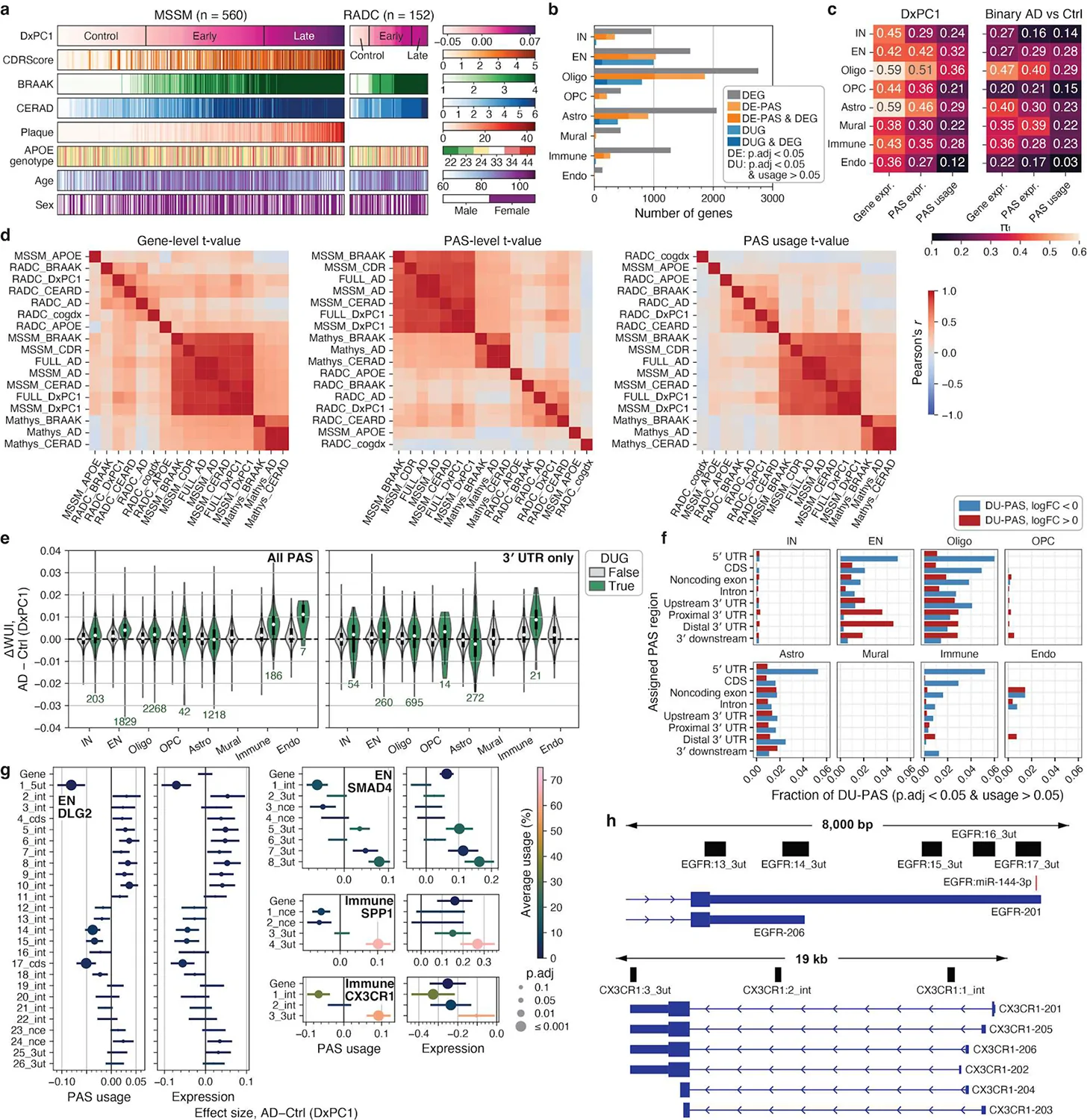
Alternative polyadenylation changes with AD severity **a**, DxPC1, the first principal component of AD severity metrics, and other AD-related clinical measures across Mount Sinai NIH Neurobiobank (MSSM) and Rush Alzheimer’s Disease Center (RADC) cohorts. **b**, Number of DEGs, genes with DE-PAS, and all-PAS DUGs, using a binary AD diagnosis. **c**, π_1_, the estimated proportion of non-null differential expression and usage events, for DxPC1 and a binary AD diagnosis. **d**, Pearson’s *r* of differential expression t-values across multiple cohorts and AD severity metrics. **e**, ΔWUI distributions for AD (DxPC1) across cell types, for all PAS (left) and 3′ UTR PAS only (right). DUG counts per cell type are noted. **f**, Fraction of DU-PAS per transcriptomic region and cell type. **g**, PAS usage and expression effect sizes (log_2_FC per 1 SD difference in DxPC1) for selected genes and cell types. **h**, Selected *EGFR* and *CX3CR1* transcripts (blue) and PAS (black).

**Extended Data Figure 6.**
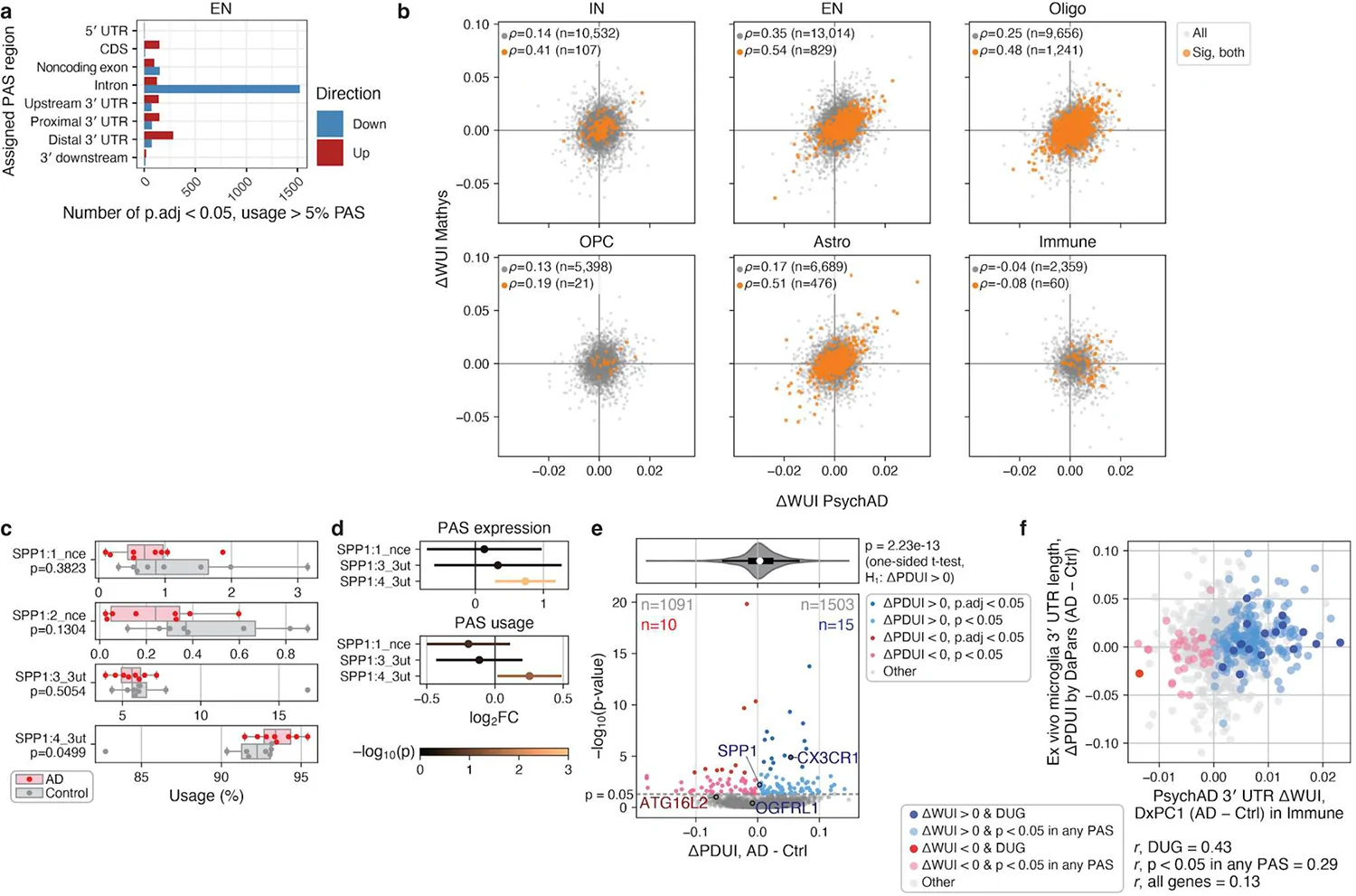
Replication of AD-associated APA changes in an external snRNA-seq dataset and in purified microglia **a**, Number of DU-PAS from the re-analysis of the Mathys dataset, in ENs only. **b**, ΔWUI correlation between PsychAD and the Mathys dataset. **c**, PAS usage of *SPP1* in purified microglia from AD and control donors, quantified by the proportion of Iso-seq reads with 3′-ends mapped within 100 bp of PAS 3′-ends. P-values were calculated by two-sided Mann-Whitney U tests. **d**, PAS expression and usage fold changes of *SPP1* between AD and control donors in purified microglia bulk RNA-seq. Transcript-level expression was aggregated to PAS-level for transcripts with 3′-ends mapped within 100 bp of PAS 3′-ends. P-values were calculated using linear mixed models with contrasts and covariates as described in Kosoy et al. (AD age model)^43^. **e**, 3′ UTR length changes in purified microglia from AD donors compared to controls^43^, measured as PDUI with associated p-values calculated by DaPars^57^. The upper violin plot shows the distribution of ΔPDUI. **f**, Correlation between 3′ UTR length changes in PsychAD snRNA-seq Immune cells (x-axis) and purified microglia bulk RNA-seq (y-axis). DUG definitions and p-values are from the PsychAD 3′ UTR APA analysis

**Extended Data Figure 7.**
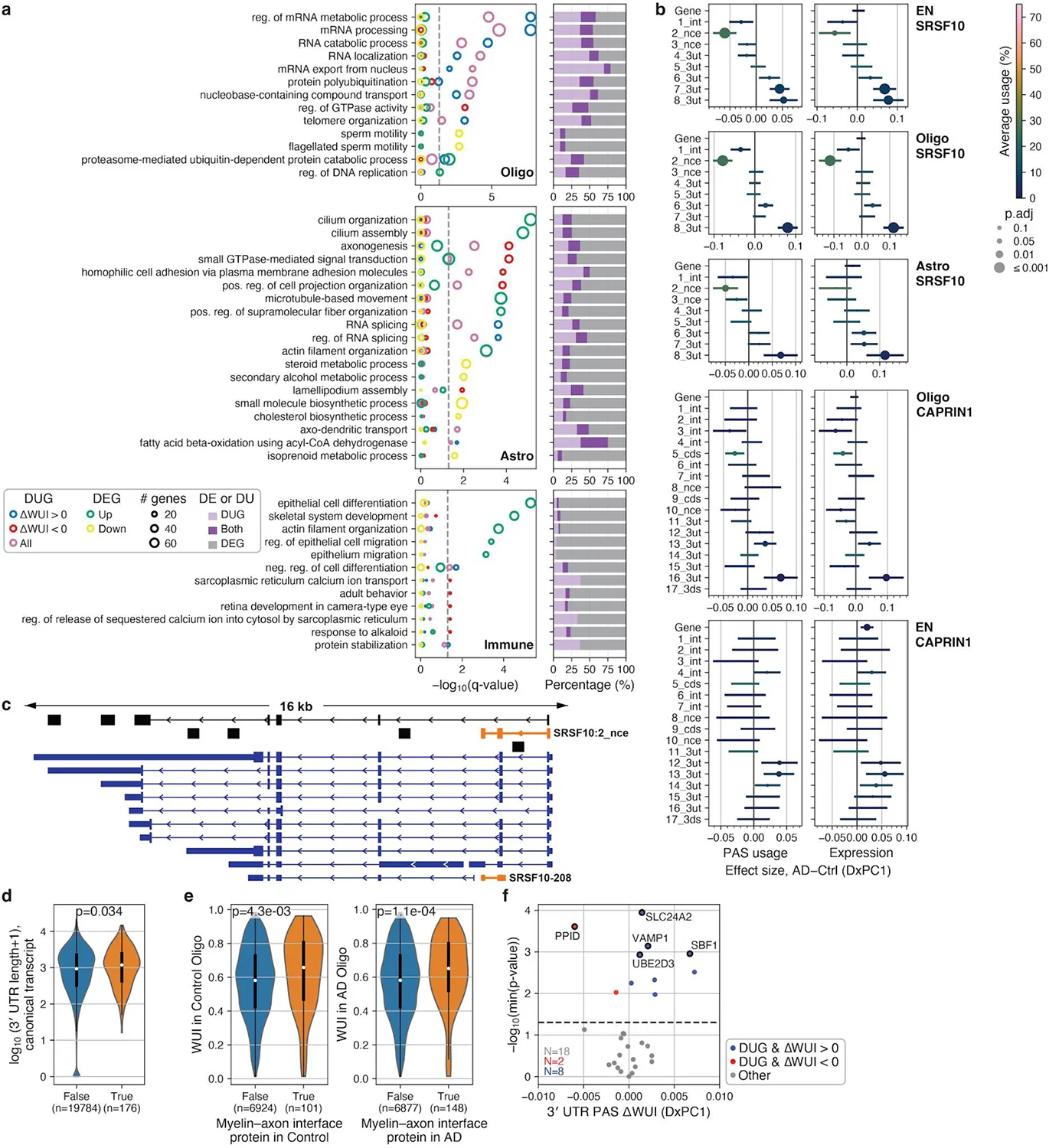
Additional analyses on alternative polyadenylation changes in AD **a**, Gene Ontology enrichment analysis for DUGs and DEGs in Oligo, Astro, and Immune. Left: q-values, right: overlap between DUG and DEG. See Fig. 5g for EN. **b**, PAS usage and expression effect sizes (log_2_FC per 1 SD difference in DxPC1) for selected genes and cell types. PAS labels indicate their order from 5′ and genomic region: int, intron; 3ut, 3′ UTR; cds, coding sequence; nce, noncoding exon. **c**, SRSF10 transcript annotation in Ensembl (blue, selected) and PAS annotation in this study (black). **d**, 3′ UTR lengths of genes encoding myelin-axon interface proteins, identified in control donors^61^. P-value by two-sided Mann-Whitney U test in this and panel **e**. 3′ UTR length from canonical transcript of each gene in Ensembl v104. **e**, Absolute WUI values for genes coding myelin–axon interface proteins, in Control and AD Oligo. **f**, Volcano plot showing ΔWUI and per gene minimum p-value of differential PAS usage, in AD Oligo.

**Extended Data Figure 8.**
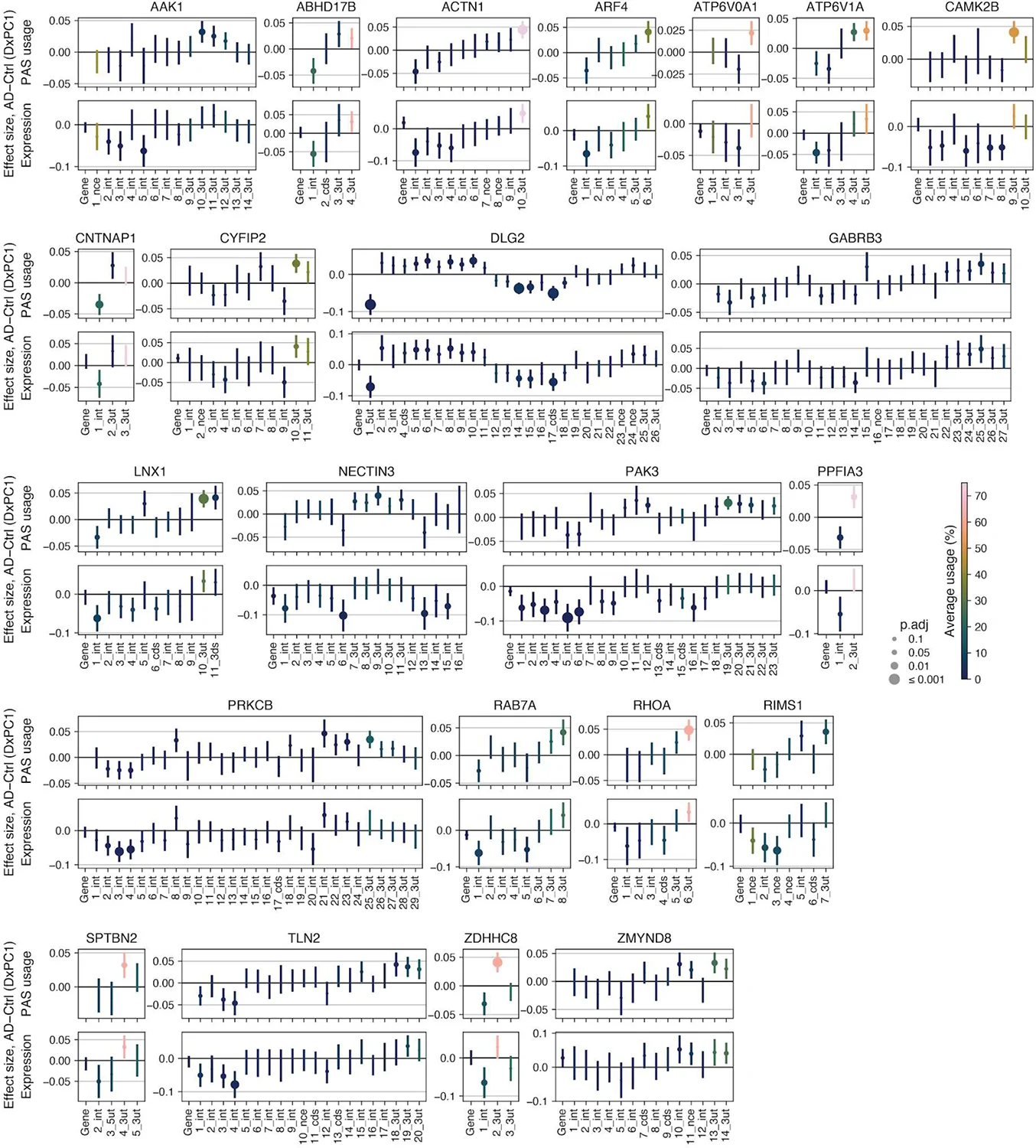
Synaptic DUGs without significant mRNA DE in AD ENs PAS usage (%) and expression effect sizes effect sizes (log_2_FC per 1 SD difference in DxPC1) for genes with differential PAS usage but no significant gene-level differential expression. PAS labels indicate order from 5′-end and genomic region: int, intron; 3ut, 3′ UTR; cds, coding sequence; nce, noncoding exon.

**Extended Data Figure 9.**
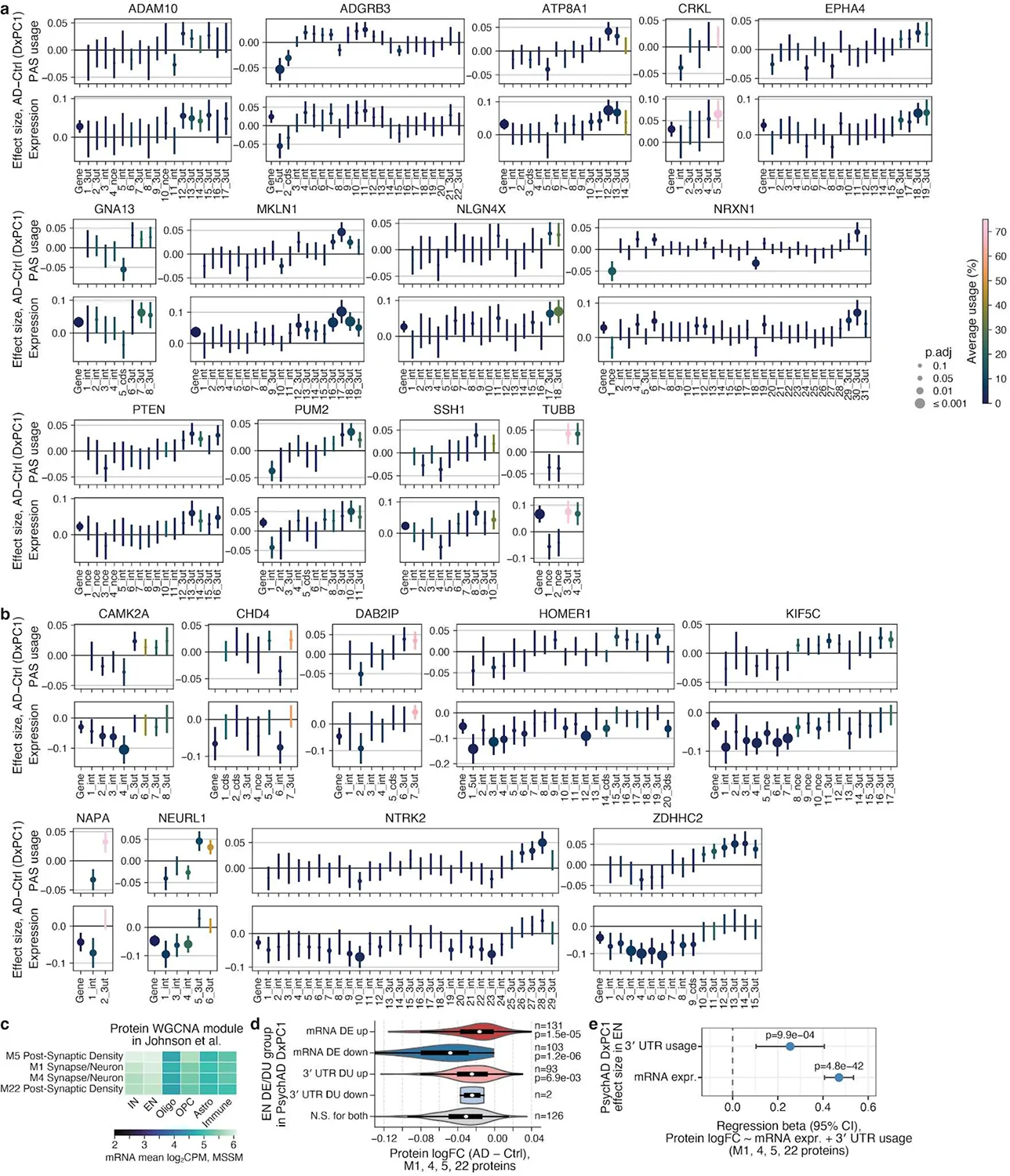
Synaptic DUGs and their protein-level associations in AD **a**, PAS usage (%) and expression effect sizes (log_2_FC per 1 SD difference in DxPC1) for genes with synaptic functions, differential PAS usage, and significant gene-level upregulation in AD. PAS labels indicate order from 5′-end and genomic region: int, intron; 3ut, 3′ UTR; cds, coding sequence; nce, noncoding exon. **b**, Same as **a** but for downregulated genes. **c**, mRNA expression of synapse-specific WGCNA modules from Johnson et al.^70^, in the PsychAD class-level cell types. **d**, AD versus control protein log fold changes of synapse-specific WGCNA modules, stratified by DxPC1 DE/DU group in the PsychAD ENs. Genes shared between the DE and DU groups were excluded. P-values were calculated by Mann–Whitney U test **e**, Coefficients and p-values from a linear model predicting protein log fold change (AD versus control) from mRNA expression and 3′ UTR PAS usage changes in AD ENs (DxPC1), fitted on synapse-specific WGCNA module genes.

**Extended Data Figure 10.**
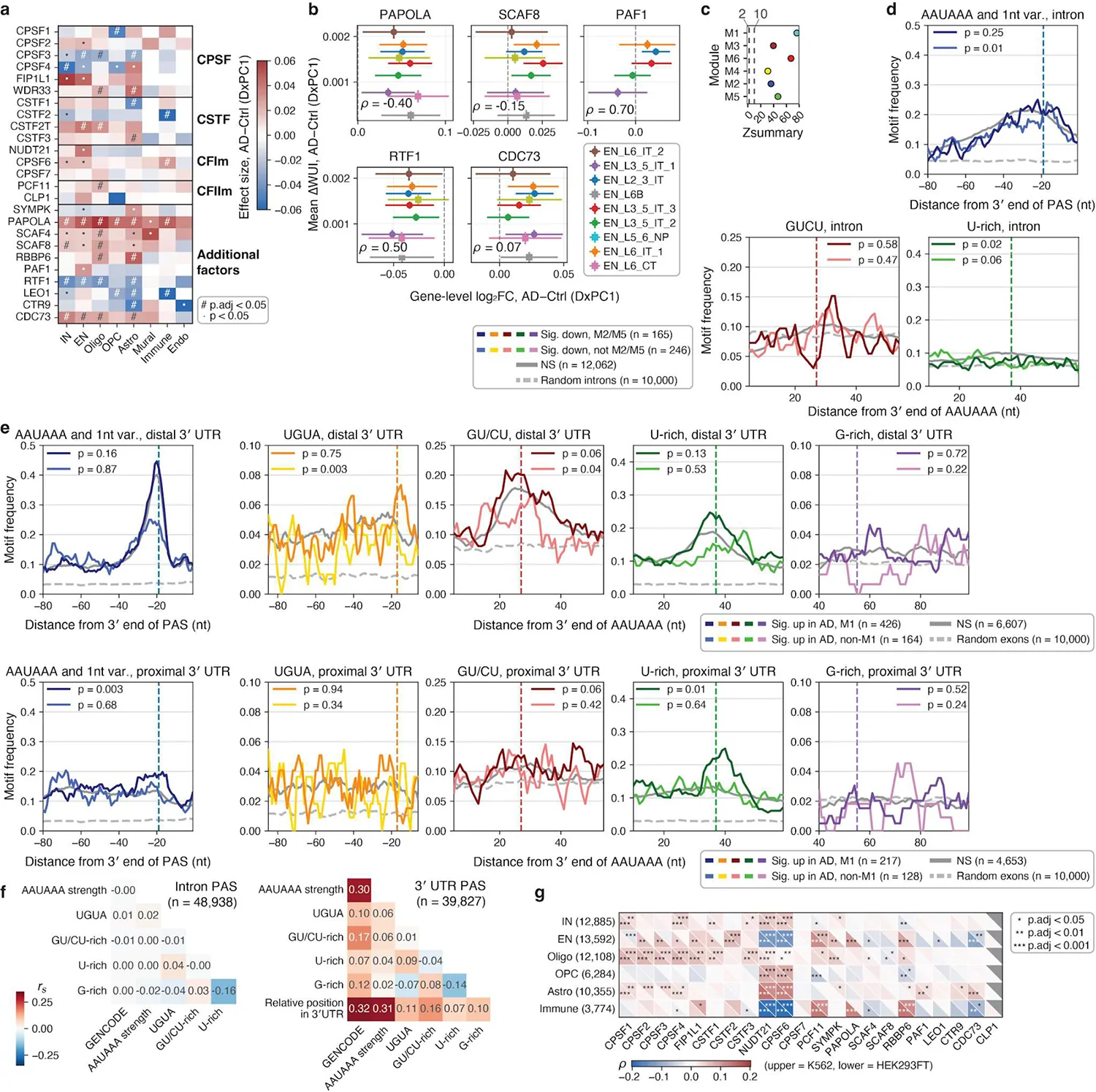
CPA factor expression, motif enrichment, and perturbation concordance **a**, DE of CPA factors in AD (DxPC1) across class-level cell types. **b**, Gene-level log fold change versus mean ΔWUI in AD (DxPC1) across EN subclasses. **c**, Preservation of the MSSM WGCNA modules (Fig. 6c) in RADC. **d**, Motif frequencies in intronic PAS, grouped by DU in AD (DxPC1) and by WGCNA module (Mann–Whitney U test). For non-AAUAAA motifs, positions are relative to the AAUAAA or 1-nt variant (excluding AAAAAA) nearest to the PAS 3′ end. Vertical lines mark optimal motif positions. Motifs shown in Fig. 6 are omitted. **e**, As in **d**, for distal (upper) and proximal (lower) 3′ UTR PAS. **f**, Spearman’s *ρ* among AAUAAA strength (2, exact match; 1, 1 nt variant except AAAAAA; 0, neither), other motif counts, PAS position, and GENCODE annotation status. **g**, Spearman’s *ρ* between PAS Δ usage (AD versus control) and Δ usage in Perturb-seq from Kowalski et al^22^. P-values were calculated by permutation (20,000 permutations, restricted to PAS within the same genomic region) and adjusted by the Benjamini–Hochberg method.

## Supplementary Material

Supplementary Files

This is a list of supplementary files associated with this preprint. Click to download.

- Suppletable6Upstreamdrivercorrelation.xlsx
- Suppletable5WGCNAofMSSMcohortDxPC1.xlsx
- Suppletable3myeloid.xlsx
- Suppletable1PASannotation.xlsx
- Suppletable4DxPC1mergedDEGandAllPASWUI.xlsx
- Suppletable2compareClusterclassGenes.xlsx

Supplementary Table 1. Polyadenylation site (PAS) annotation used in this study

Supplementary Table 2. Cell-type-specific gene expression, Δweighted usage index (WUI), and ΔWUI clustering results across major cell classes.

Supplementary Table 3. Differential gene expression, PAS expression, and PAS usage analysis across brain myeloid cell subtypes

Supplementary Table 4. Differential gene expression and alternative polyadenylation changes in Alzheimer’s disease

Supplementary Table 5. WGCNA module assignment and membership for PAS usage fold changes from the DxPC1 test in the MSSM cohort

Supplementary Table 6. Correlations among AD differential expression, WGCNA module membership, and APA in ENs

## Figures and Tables

**Figure 1.**
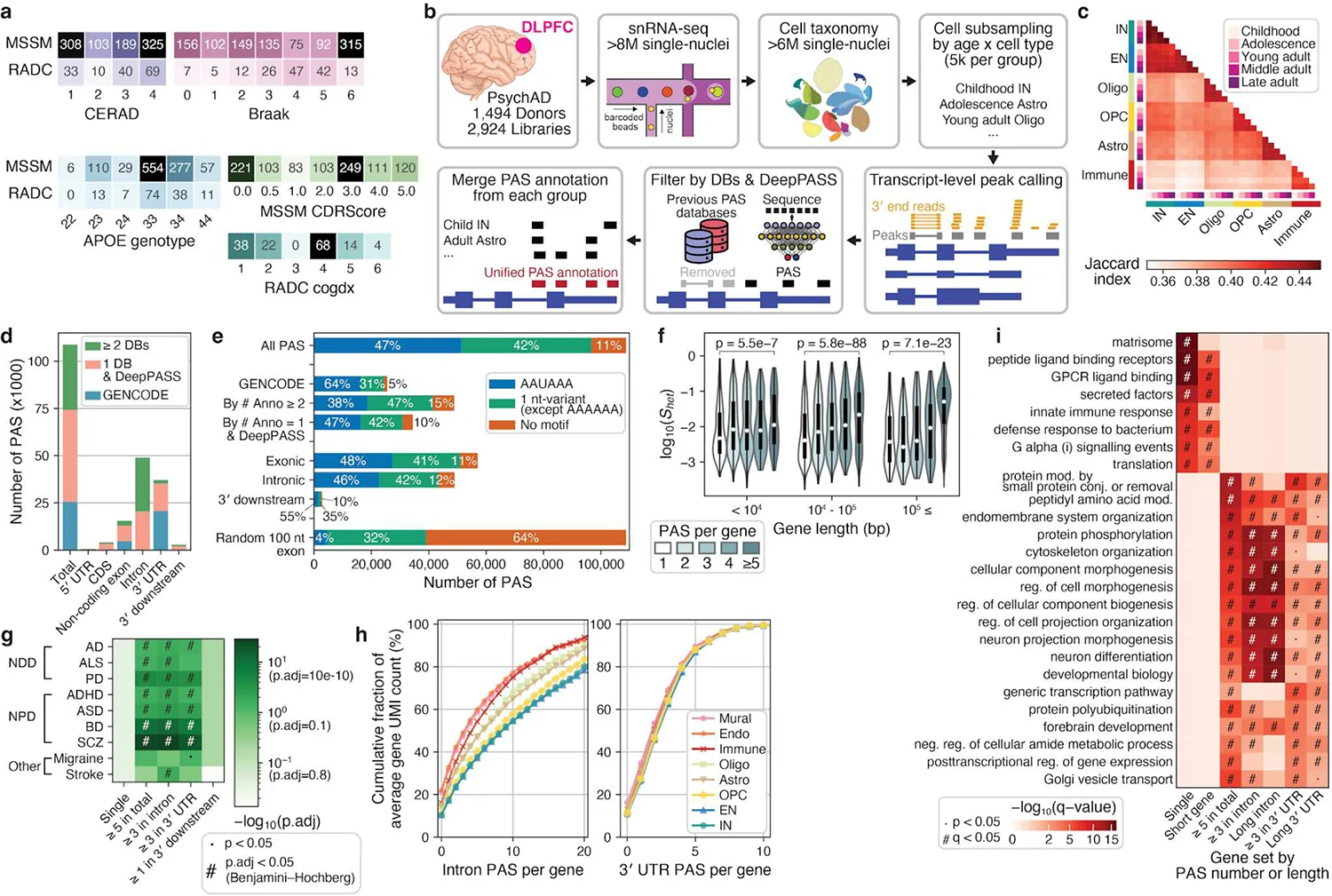
Comprehensive polyadenylation site annotation in the human dorsolateral prefrontal cortex **a**, Number of donors stratified by tissue source, neuropathological assessment, and clinical dementia status. **b**, Schematic overview of polyadenylation site (PAS) annotation pipeline. **c**, Jaccard index heatmap showing PAS annotation overlap between different age groups and cell types. **d**, Number of annotated PAS by transcriptomic region and annotation source. **e**, Number and percentage of PAS containing the canonical polyadenylation signal motif (AAUAAA), one-nucleotide variants, and neither, within −90 to +10 bp of the PAS 3′-end. **f**, Evolutionary constraint of genes measured as S_het_ scores, grouped by PAS count and gene length. P-values were calculated using two-sided Mann-Whitney U tests. Inner boxplots show the median, first and third quartiles (box), and 1.5× the interquartile range (whiskers); this definition applies to all subsequent figures. **g**, MAGMA gene-set enrichment analysis testing associations between PAS characteristics and genetic risk for brain disorders. Abbreviations: Alzheimer’s disease (AD), amyotrophic lateral sclerosis (ALS), Parkinson’s disease (PD), attention-deficit/hyperactivity disorder (ADHD), autism spectrum disorder (ASD), bipolar disorder (BD), and schizophrenia (SCZ). **h**, Cumulative fraction of Unique Molecular Identifier (UMI) counts per gene as a function of the number of intronic or 3′ UTR PAS. **i**, MSigDB pathway enrichment for gene sets stratified by PAS count or gene length. Short or long genes were defined as the 5,000 shortest and 5,000 longest genes, respectively.

**Figure 2.**
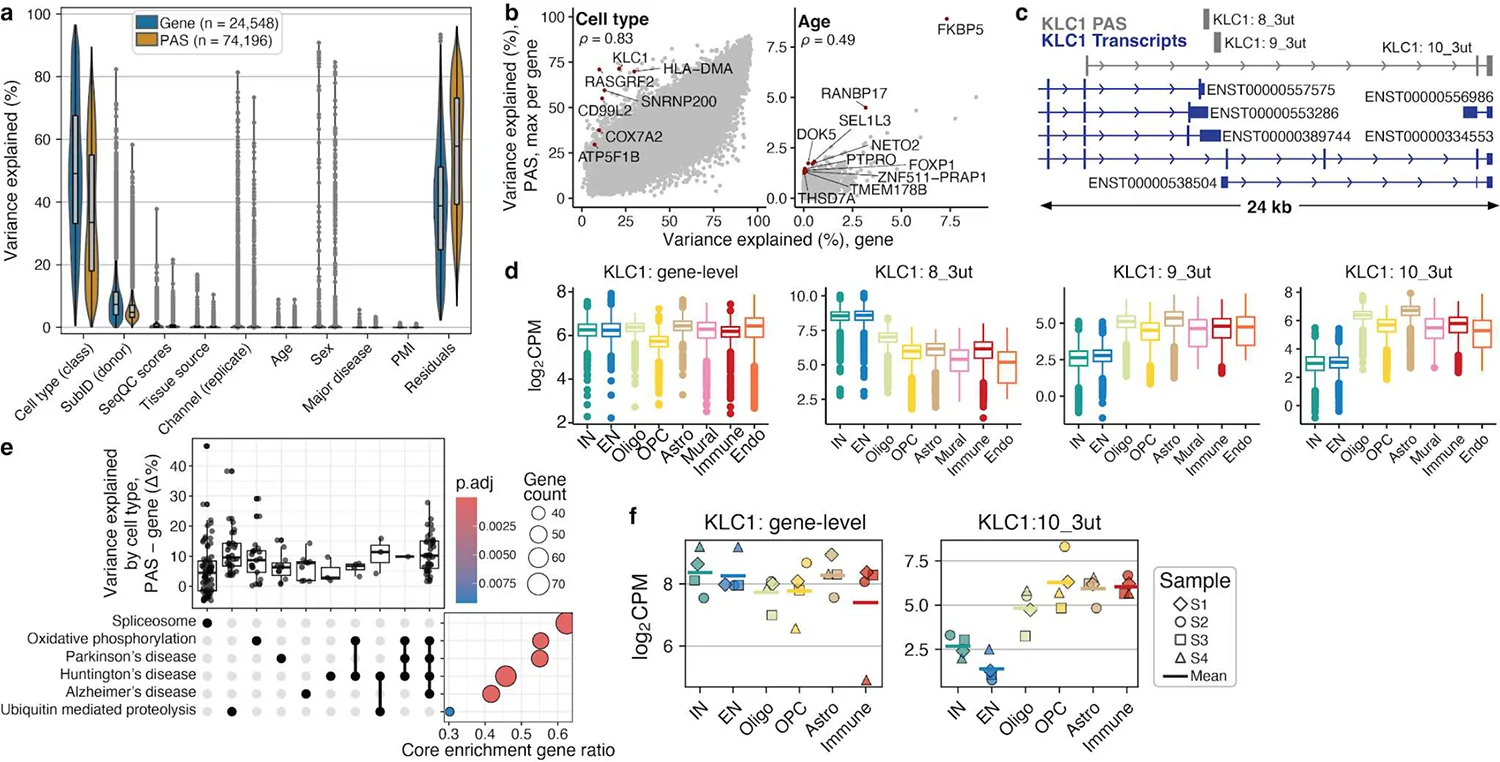
Sources of variation in gene- and PAS-level expression **a**, Variance partition analysis of gene- and PAS-level expression. SeqQC denotes the summed variance explained by sequencing-derived QC metrics, including the number of detected genes, mitochondrial and ribosomal read fractions, GC content and 5′-to-3′ bias. **b**, Expression variance explained by cell type (left) and age (right), at PAS-level versus gene-level. The maximum explained variance across all PAS of each gene is shown, and each dot represents one gene. **c**, *KLC1* transcripts (selected from Ensembl, blue) and PAS (this study, gray), from the 3′-most 24 kb. **d**, *KLC1* expression at gene- and PAS-level. log_2_CPM values in this panel and panel **f** are normalized per replicate and cell type. **e**, KEGG pathway enrichment of genes with high cell-type-specific PAS expression, by Gene Set Enrichment Analysis. Upper: Difference in variance explained by cell type (PAS − gene, Δ percentage). Lower left: Gene overlap shown as an upset plot. Right: Significance of enrichment. **f**, Long-read RNA-seq results for *KLC1*, by Multiplexed Arrays Sequencing.

**Figure 3.**
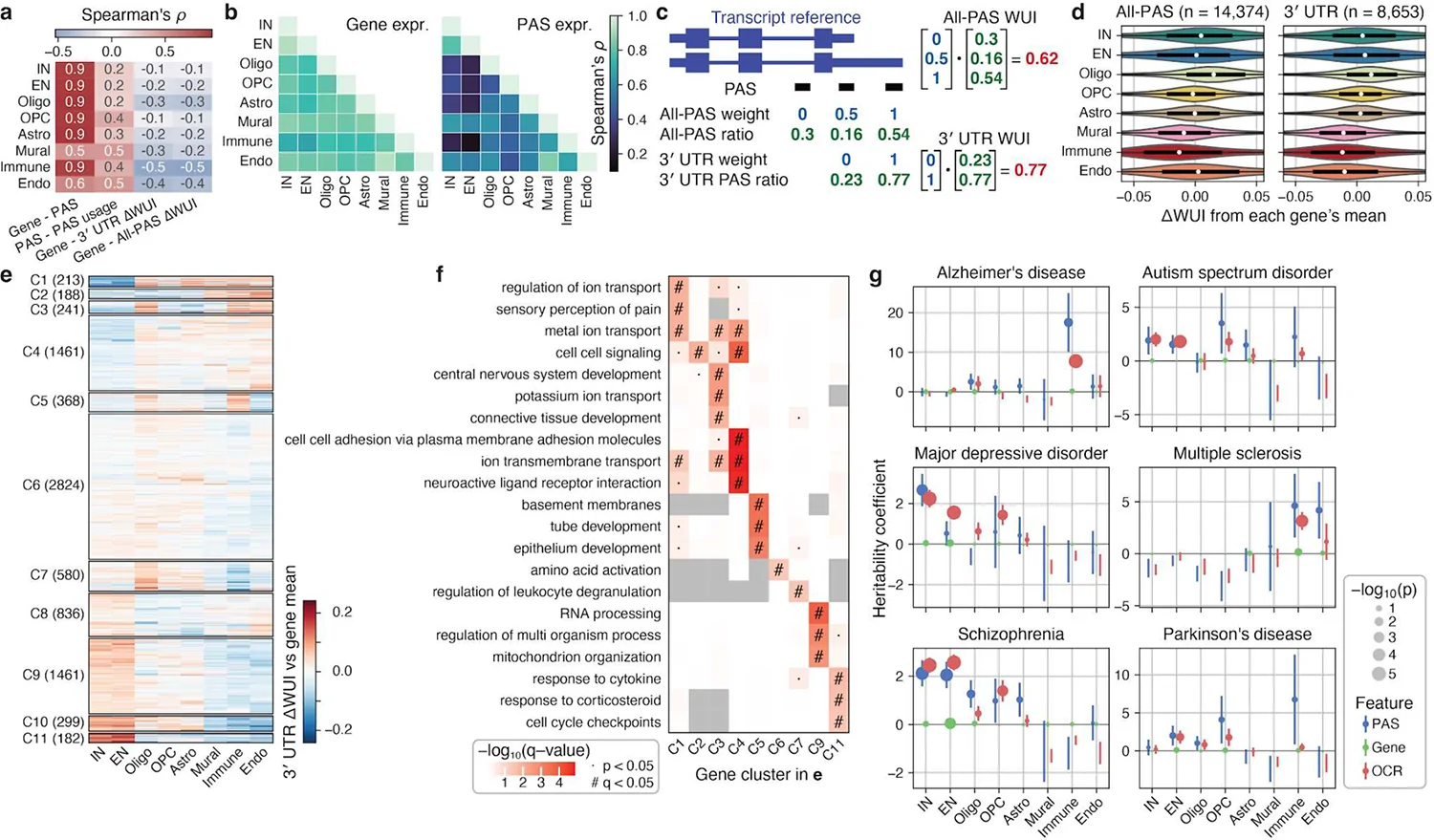
Alternative polyadenylation across major brain cell types **a**, Correlation between log fold changes (log FC) of gene expression, PAS expression, PAS usage, and ΔWUI metrics for each cell type versus all others. **b**, Pairwise correlation of gene- and PAS-level expression (log_2_CPM) between class-level cell types. **c**, Schematic of Weighted Usage Index (WUI) calculation. WUI quantifies the relative 3′-end position of a gene. All-PAS WUI measures general 3′-end lengthening or shortening, whereas 3′ UTR WUI specifically measures 3′ UTR length variation. **d**, Distribution of all-PAS and 3′ UTR ΔWUI for each cell type, shown as differences from each gene’s mean. **e**, K-means clustering of genes for 3′ UTR ΔWUI patterns across cell types. Numbers indicate genes per cluster. **f**, MSigDB pathway enrichment analysis of gene clusters in (**e**). **g**, Linkage Disequilibrium Score Regression (LDSC) analysis showing heritability enrichment for cell-type-specific PAS, genes, and open chromatin regions (OCRs). Cell-type-specific PAS and genes were defined by log_2_FC > 1 and p.adj < 0.01, and OCRs by log_2_FC > 1 and p.adj < 0.05. Error bars indicate standard error.

**Figure 4.**
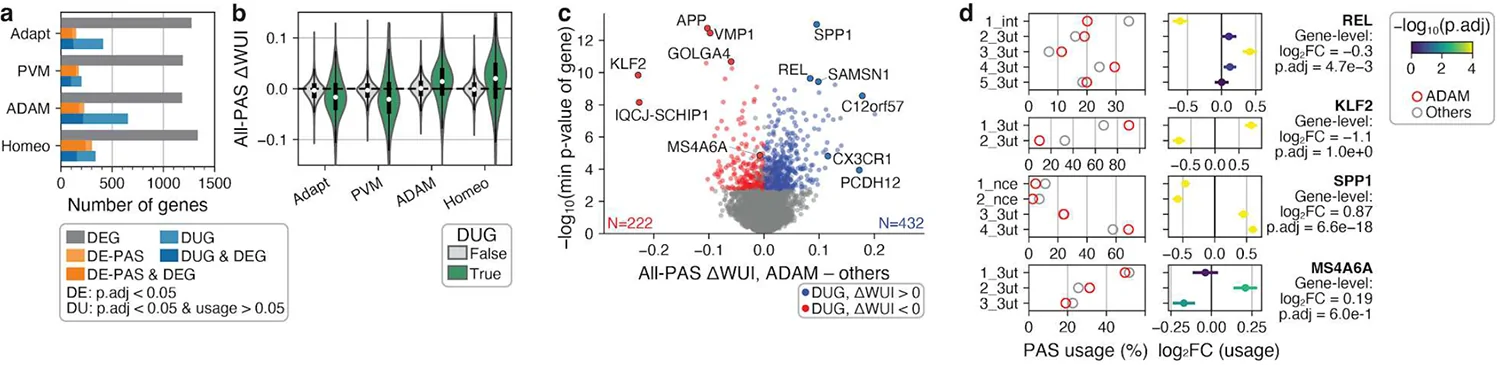
Alternative polyadenylation across microglial states **a**, Number of differentially expressed genes (DEGs), genes with any differentially expressed PAS (DE-PAS), or genes with any differential usage PAS (DUGs) across brain myeloid subtypes. **b**, ΔWUI of brain myeloid subtypes, from each subtype versus all others. **c**, All-PAS ΔWUI (x) and minimum PAS-level p-value per gene (y), for AD-associated microglia (ADAM) versus other brain myeloid subtypes. For other subtypes, see Extended Data Fig. 4c. **d**, PAS usage and usage log FC, between ADAM and other brain myeloid subtypes. Error bars indicate 95% confidence intervals. For PAS and transcript structures, see Extended Data Fig. 4e.

**Figure 5.**
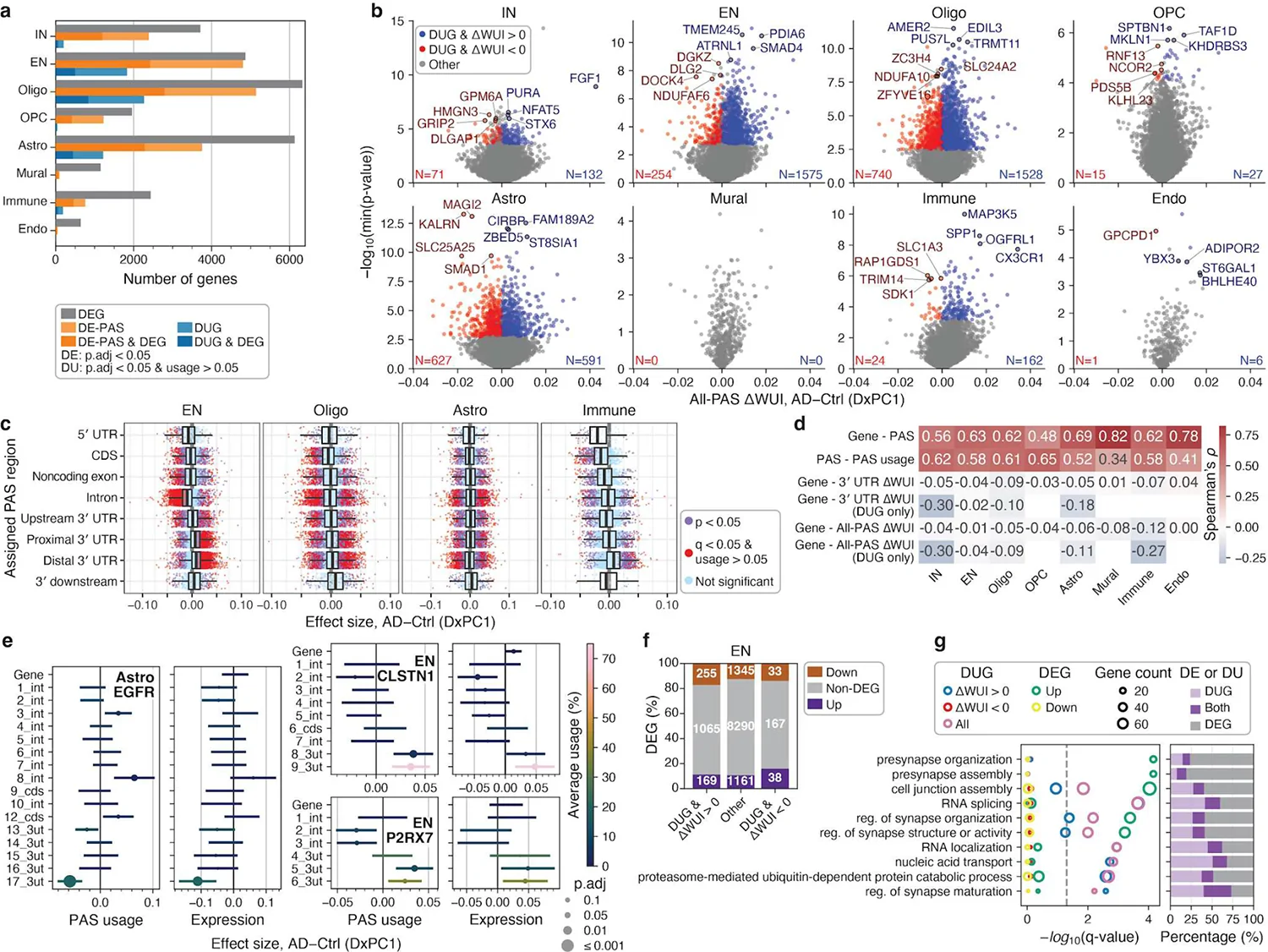
Cell-type-specific alternative polyadenylation changes in AD **a**, Number of DEGs, genes with DE-PAS, and DUGs by DxPC1-based analysis. **b**, All-PAS ΔWUI (per 1 SD difference in DxPC1) and minimum PAS-level p-value per gene for each cell type. Numbers indicate DUG counts in each ΔWUI direction. **c**, Usage effect size (log_2_FC per 1 SD difference in DxPC1) of transcriptomic regions in EN, Oligo, Astro, and Immune cell types. To quantify regional usage, UMI counts of all PAS falling in the same region were summed for each gene. **d**, Correlation between gene-level and PAS-level differential expression and usage metrics in AD. Comparisons with <50 genes were omitted. **e**, PAS usage and expression effect size (log FC per 1 SD difference in DxPC1) for selected genes and cell types. Error bars indicate 95% confidence intervals. PAS labels indicate their order from 5′ and genomic region: int, intron; 3ut, 3′ UTR; cds, coding sequence; nce, noncoding exon. **f**, Number and proportion of upregulated, downregulated, and non-significant DEGs among genes stratified by DUG status and ΔWUI direction in ENs. **g**, Gene Ontology enrichment analysis for DUGs and DEGs in ENs, showing q-values (left) and the overlap percentage between DUGs and DEGs (right). See Extended Data Fig. 7a for other cell types.

**Figure 6.**
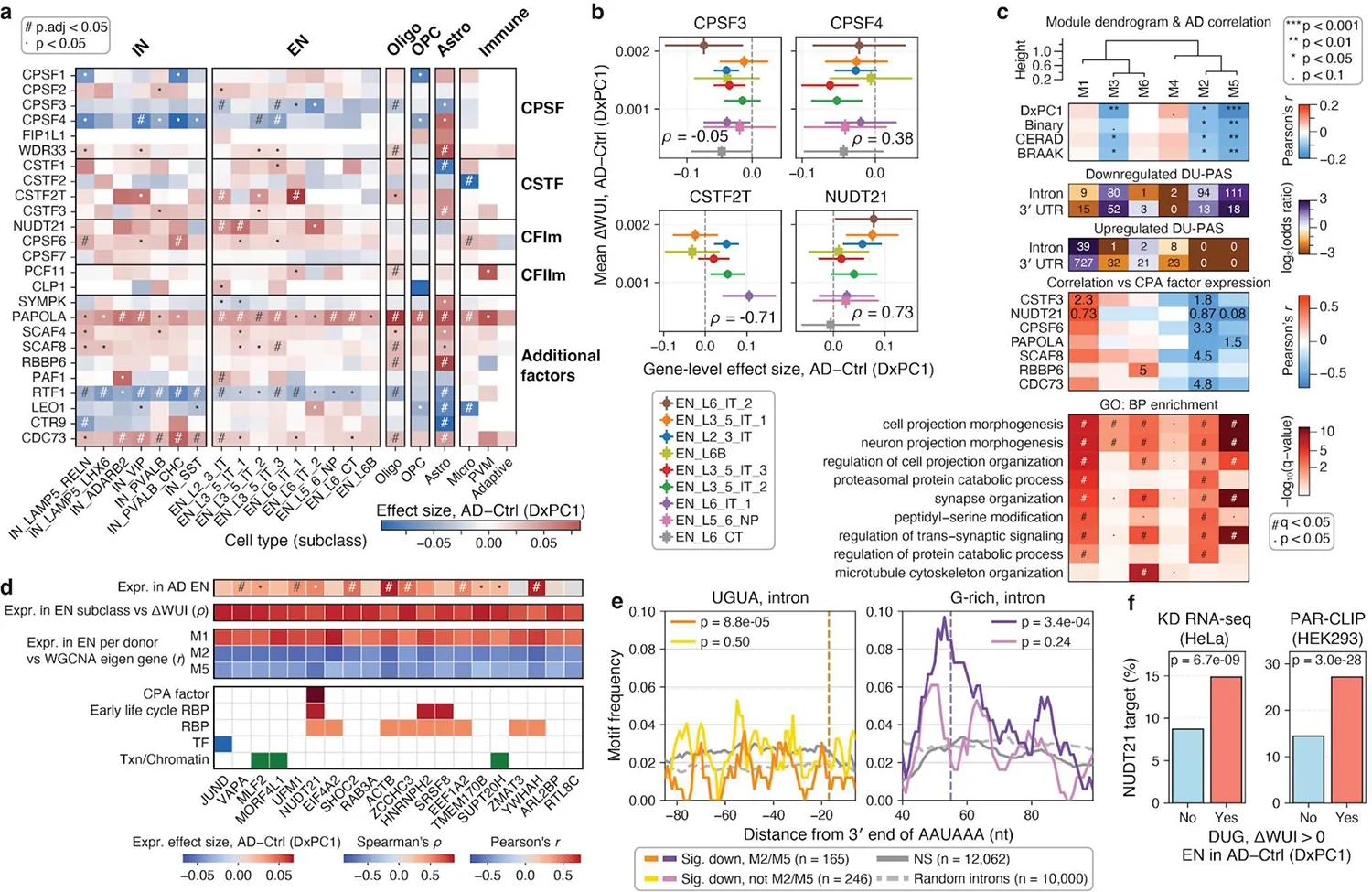
Cleavage and polyadenylation factor expression and their association with APA in AD **a**, Differential expression of major and additional cleavage and polyadenylation (CPA) factors in AD (DxPC1), across cell type subclasses. **b**, Correlation between gene expression and mean ΔWUI in AD, across EN subclasses. **c**, WGCNA analysis of PAS usage in the MSSM cohort. Top: module-trait correlations with DxPC1, binary AD diagnosis, and neuropathological metrics. Second and third rows: enrichment of DU-PAS with up (upper) or downregulation (lower) in AD (DxPC1); numbers indicate PAS counts. Fourth row: CPA factors showing module eigengene r^2^ in the top 5% amonĝ16k tested genes, labeled with r^2^ percentile. Bottom: Top 3 enriched GO terms per module. **d**, Heatmap of mRNA DE in AD ENs (upper), cell-type-level and donor-level correlations between gene expression and APA (middle two), and known function of gene (lower). Genes with the lowest summed correlation ranks are shown. **e**, UGUA and G-rich motif frequencies in intronic PAS, grouped by DU in AD (DxPC1) and by WGCNA module (Mann–Whitney U test on counts within the window). Vertical lines show optimal position of each motif. **f**, Overlap between *NUDT21* target genes from previous studies and 3′-lengthened DUGs in AD ENs.

## Data Availability

A cleaned version of the computational pipeline for PAS annotation, quantification, and statistical testing is available at https://github.com/DiseaseNeuroGenomics/PsychAD-APA. The full analysis code and differential expression/usage test results for all genes are archived on Zenodo (https://doi.org/10.5281/zenodo.22260821). Raw PAS-level count data (syn74739724) and donor-level DxPC1 scores (syn74913889) are available via the Synapse platform (https://www.synapse.org/) and the AD Knowledge Portal (https://adknowledgeportal.org). Long-read sequencing data will be deposited in a controlled-access database upon publication; in the meantime, the data are available to reviewers upon request.
